# The lateral batting backlift technique: is it a contributing factor to success for professional cricket players at the highest level?

**DOI:** 10.17159/2078-516X/2019/v31i1a5460

**Published:** 2019-01-01

**Authors:** M H Noorbhai, T D Noakes

**Affiliations:** 1 Department of Human Movement Science, Faculty of Health Sciences, University of Fort Hare, Alice, South Africa; 2 Division of Exercise Science and Sports Medicine, Department of Human Biology, Faculty of Health Sciences, University of Cape Town, Cape Town, South Africa

**Keywords:** video analysis, biomechanics, coaching, optimising performance

## Abstract

**Background:**

This study aimed primarily to investigate the lateral batting backlift technique (LBBT) among semi-professional, professional and current international cricket players. A key question was to investigate whether this technique is a factor that contributes to success for cricket players at the highest levels of the game.

**Methods:**

The participants in this study’s sample (n = 130) were South African semi-professional players (SP) (n = 69), professional players (PP) (n = 49) and South African international professional players (SAI) (n = 12). Biomechanical and video analyses were performed on all the participating groups. Classifiers were utilised to identify the batting backlift technique type (BBTT) employed by all batsmen. All statistics and wagon wheels (scoring areas of the batsmen on a cricket field) were sourced online. A Pearson’s Chi-squared test, Student T-test, one-way analysis of variance and T-test were performed in this study. All analyses were performed using R (R Core Team) at a significance level of α = 0.05.

**Results:**

This study found that a LBBT is more common at the highest levels of batsmanship with batsmen at the various levels of cricket having percentages of the LBBT as follows: SP = 37%; PP = 38%; SAI = 75%; p = 0.001. There was also a noticeably higher difference in the highest scores and career averages between all groups of players, as well as batsmen who either use a straight batting backlift technique (SBBT) or a LBBT. This study also found that SAI batsmen who used the LBBT were more proficient at scoring runs in various areas around the cricket field (according to the wagon wheel analysis).

**Conclusion:**

This study found that a LBBT is a contributing factor for success regarding players wanting to play cricket at the highest levels. Cricket coaches should also pay attention to the direction of the backlift with players, especially when correlating it to various scoring areas on the cricket field. Further in-depth research is required to fully investigate the change in batting backlift techniques among cricket players over a long-term period.

The mechanics of the backlift in cricket batting are poorly understood.^[1]^ Qualitative biomechanical analyses of movement in sports are key to its investigation.^[2]^ Such a mode of investigation can provide important insights into the biomechanics of technique in sports.^[3]^ Cricket batting is complex with different variables such as the grip, stance, initial movement, backlift, downswing and follow through.^[4]^ An important component of the overall batting technique is the backlift, a technical component of batting that has defied the traditional attempt to constrain its motion to the linear plane.^[4,5]^ The most proficient run-scorers of the game lift the bat from the region of the slips, often causing the downswing path of the bat to deviate from its upswing. Devising a qualitative biomechanics model of the backlift could therefore do much to probe its underlying mechanics.^[6]^

Examinations of the backlift of the bat provide an interesting insight into how skilled batters achieve control of the bat to effectively and efficiently swing their arms to successfully strike a ball. Many batters have been observed to adopt a backlift that is diverted away from their body, rather than positioning their bat directly behind them as is commonly advocated in the coaching literature.^[5]^ This is contrary to what may be logically expected as the most effective means of preparing for a straight and efficient downswing.^[5,7]^ It was also found that angling of the backlift away from the body was common, and was similar for all skilled and lesser-skilled batters. It has been proposed that this angle may provide a comfortable position for the batters to place their hands in preparation for the subsequent downswing, allowing for a possible more ‘rotary’ movement of the wrists by which the bat’s backswing and downswing can be performed in a continuous motion.^[5]^

Research conducted in Australia by Stuelcken et al. ^[7]^ on international batsmen (n = 9) was one of few studies that demonstrated findings of the backlift in cricket batting. The study showed that path tracings of the bat indicated a distinctive loop, which was unexpected.^[7]^ There was no clear evidence provided by the authors to explain the cause of this significant loop, aside from the fact that a greater diversity of strokes would be a possible outcome as batsmen would get used to hitting the ball in this way. In addition, it was found that the path of the bat deviated laterally from the mean alignment of the shoulders, reaching an average maximum angle in the transverse plane of 47° (after the batsmen initiated the backlift). The study then indicated how this angle was reduced by a mean of 23° at the top of the backlift, which showed that the position of the bat was increasingly lateral from an alignment that would enable the required bat plane to drive to the offside.^[8]^ Stuelcken et al.^[7]^ also proposed that batsmen manoeuver their bat by using their wrists as levers to position the bat close to the body’s centre of mass. This may help to keep the bat’s centre of mass close to the batter’s base of support, and ultimately allow a later downswing, thereby helping to overcome the temporal constraints inherent in batting. If the wrists were to be moved away from the body in the bat’s backswing, more energy and time would be required to produce the backswing and downswing respectively.^[7,8]^ If the wrists are kept close to the body, the batter is afforded a mechanical advantage as the moment of inertia required to move the bat at a given velocity is reduced. This decreases the amount of muscular effort required to play a stroke, allowing the bat to travel through a smaller arc to enable its faster movements.^[7,8]^

Recently, Noorbhai and Noakes^[8]^ followed on from the above study which had shown that a vast majority of successful batsmen (77%) in the last century had used a lateral batting backlift technique (LBBT). It was also investigated that the LBBT is a key contributing factor to the success of the overall cricket batting technique.^[8]^ The LBBT is one in which the bat is lifted laterally in the direction of second slip or gully. Therefore, the face of the bat is directed towards point or the offside. By contrast, the backlift, where the bat is lifted towards the stumps or first slip and the face of the bat points towards the wicket-keeper or the ground, is known as the straight batting backlift technique (SBBT).^[8]^

Expert coaches have frequently supported the notion that there is no necessarily ‘right’ or ‘wrong’ way to bat, and that many of the greatest players have exhibited techniques not necessarily commensurate with those recommended in coaching manuals.^[9]^ For example, Sir Donald Bradman (widely considered as the greatest batter of all time) exhibited a highly unique ‘rotary’ technique, which is contrary to coaching conventions, and is yet to be replicated.^[8,9]^ In modern coaching manuals (those published after 2009), it has become an acceptable norm for batsmen to lift the bat in the direction of the slips. A mixed-methods study conducted among 161 coaches around the world showed that most of them (83%) coach the SBBT technique as opposed to the LBBT at various proficiency levels of the game.^[9]^ With regards to the higher levels of cricket, most coaches understand the potential value of the LBBT but have challenges coaching it. ^[9]^

As a previous study by these authors had analysed successful batsmen only at the highest international level ^[8]^, this present study attempts to investigate the batting backlift technique (BBT) among semi-professional, professional and current international cricket players. The findings of the previous study suggested that the LBBT is a likely contributing factor to effective batsmanship. Therefore, a key question in this present study was to investigate to what extent batters at the lower levels of the game use the LBBT. These authors’ hypothesis suggests that the LBBT acts as a selective factor among proficient batters at the highest levels of cricket.

Since the backlift direction can be readily detected with qualitative observations from the direction in which the toe of the bat is pointing, and since there are additional gaps to explore regarding the LBBT as highlighted above, these authors considered it feasible to investigate the following question: is a LBBT used more frequently by batters at the highest levels of the game, compared to batters at lower levels?

As previously mentioned, the authors hypothesised that the LBBT is a contributing factor for cricket players wanting to play cricket at the highest levels of the game.

## Methods

### Study design

This was a cross-sectional research study in which analytical and qualitative biomechanical research methods were employed.

### Ethical considerations

Ethical approval for the study was granted by the Human Research Ethics Committee of the University of Cape Town (HREC: 586/2014). All participants provided signed consent prior to participating in the study. This study conforms to the World Medical Association Declaration of Helsinki on Ethical Principles for Research Involving Human Subjects.

### Participants

**Fig. 1:**
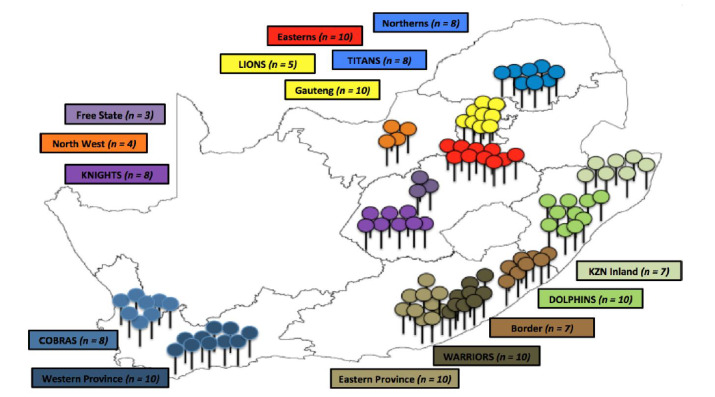
Semi-professional and professional cricket players from South Africa (n = 118). Semi-professional teams include Western Province, Eastern Province, Border, KwaZulu-Natal Inland, Northerns, Gauteng, Easterns, North West and Free State. Professional teams include Cobras, Warriors, Dolphins, Titans, Lions and Knights.

Participants (n = 130) were South African semi-professional players (SP) (n = 69), professional players (PP) (n = 49) (Fig. 1) and South African international professional players (SAI) (n = 12) who played in the Cricket South Africa (CSA) domestic competitions and International Cricket Council (ICC) fixtures respectively during the 2015/2016 cricket season. The sample group of SP is reflective of nearly 50% of the total population sample of the 13 semi-professional teams with approximately 143 players in South Africa, while the PP group (n = 49) is part of the six franchise teams in South Africa, which is indicative of 74% of the total sample of approximately 66 players. All players either represented their provincial, franchise or national team(s).

### Study procedure

Various types of deliveries (n = 6; two short deliveries, two good length deliveries, two full deliveries, either pitched on middle leg or outside off stump) were analysed from the SP and PP when they faced a fast, fast-medium or spin bowler. Any deliveries that were determined as wide, no-ball or a full toss were excluded from this analysis. Participants were required to bat using their usual batting technique in either a match or practice situation. For the SAI group, the deliveries were randomly chosen. Similarly, any deliveries that were determined as wide, no-ball or a full toss were also excluded from this analysis.

The researchers had considered the use of the bowling machine to ensure standardisation during the study. However, the objective of the study was to mimic a match situation (through practices in the nets or match practices) and an environment where various players had bowled to the batsmen. Legitimate deliveries by the bowlers were not permutated, allowing for the measures and responses from the batsmen to be consistent and accurate. All six deliveries per batsman were analysed and the still frame that best represented the player based on how the various deliveries were faced. The batsmen were used in the figure descriptions to determine the backlift type.

For the SAI players, video footage was obtained via YouTube (http://www.youtube.com), since it was challenging to recruit the South African international cricket team while they were on tour overseas.

### Biomechanical and video analyses

**Fig. 2:**
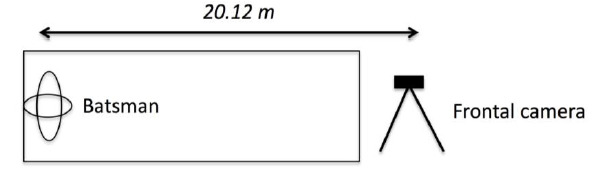
Camera setup for the analysis in the frontal view

Biomechanical and video analyses were performed on all participating groups. These included the measurement of a photo sequence with drawing tools, and a static angle range calculation of the batsman’s technique utilising the Kinovea^TM^ (Kinovea by Joan Charmant, version 0.8.15) software package, in conjunction with a virtual protractor to ensure further reliability of the angle ranges. The analyses were done similarly to those in other studies^[7,8]^ whereby the initial movement of the batsman was determined from the first frame before the initiation of the backlift, while initial movement patterns were assessed qualitatively by viewing the footage. The backlift represented the period from the initiation of the backlift to the maximum vertical displacement of the toe of the bat. The video frame was also selected immediately before the bowler released the ball. These frames were then used to determine the type of batting backlift technique for each type of delivery bowled. Variables of interest included the direction of the backlift and the direction of the face of the bat during the backlift from a Canon LEGRIA HF R506 HD Camcorder^TM^ video camera attached to a laptop computer. An external hard drive from the video camera was inserted into the laptop for further usage of the software. The frontal camera was situated 20 m away from the participants (for the SP and PP only) and in the line of where the bowler released the ball, just behind the bowler facing the batsman (Fig. 2).

### Classifiers of the backlift

Classifiers were utilised to identify the type of batting backlift technique employed by all batsmen. These classifiers were coded respectively as 1 (bat face facing straight back and towards the wicket-keeper or the ground), 2 (bat face facing first or second slip), and 3 (bat face towards gully or point). If the bat is directed fairly straight back or towards the slips/gully regions but has an open face, it is classified as classifier 3. Angle ranges were conceptualised to determine these classifiers (1: between 0° – 25°), (2: between 25° – 45°), (3: between 45° – 80°).

**Fig. 3:**
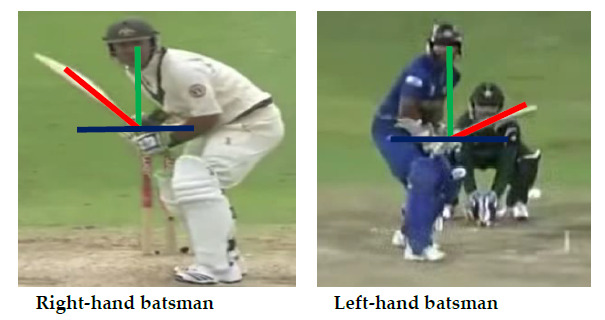
Lines and vectors drawn to depict the angle of the backlift (Adapted from: Noorbhai et al., 2016^[12]^). Note: Both these batsmen are using the LBBT

For the purpose of this study, the toe of the bat is defined as the vector orthogonal to the toe being the pointer.^[10]^ This strengthens the validity and reliability of the analysis as the backlift can be readily detected and analysed at different positions and time points in the backlift.^[11]^ Drawing a vector is a common approach in defining the toe of the bat and how it will point in a particular direction.^[2]^ Lines and vectors were drawn (1) vertically from the head to the hands (green line), (2) a line drawn horizontally to show where the hands rest (blue line), and (3) a line drawn obliquely to show the direction of the bat during the backlift (red line) (Fig. 3). The lines that were used on the batsmen were done on an individual basis as each batsman’s hands (where they rest) and the vertical line are different. The horizontal line is the starting point of where the batsman’s hands rest. As such, if the vertical line meets at one batsman’s left eye or the other batsman’s right eye, it is of no consequence, as in each case the player’s head rests in different proportions to where their hands rest. The oblique line started from the top of the bat towards the toe of the bat which depicted the angle range. The still photo of the batsman was analysed while the ball had just been released from the bowler. These lines create an angle range to show how far away the bat is from the body in the frontal plane and how much rotation is performed before the bat makes contact with the ball. The researcher accounted for perspective error by limiting the type of videos observed, as well as including horizontal lines in the background in a separate document for analysis.

### Search strategy and sources for players’ career statistics and wagon wheels

Cricinfo (http://www.espncricinfo.com) was used to retrieve the career statistics of each player (matches played, highest score, career runs scored, averages and strike rates). South African domestic players’ statistics were also sourced from their first-class (three- or four-day games) and List A (one-day games) results.

In addition, wagon wheels of the SAI were also sourced via Cricinfo to determine the areas on the cricket field where the batsmen were scoring their runs and to correlate those areas with their batting backlift technique. Wagon wheels and video footage of the SAI were obtained from a player’s highest score in a test or ODI match. Wagon wheels of the SP and PP were not available. However, the picture frames from the video footage of the SP (Western Province, Eastern Province, Border, KwaZulu-Natal Inland, Northerns, Gauteng, Easterns, North West and Free State) and PP (Cobras, Warriors, Dolphins, Titans, Lions and Knights) were used to analyse the batting backlift technique of the players.

### Quantitative data analysis

A Pearson’s Chi-squared test was performed to determine whether percentages of batsmen using a LBBT differed between the levels of professional cricket. The Student’s T-test was used to compare highest scores, career averages and strike rates between batsmen with a LBBT and SBBT, and batsmen in each population group (SP, PP and SAI), respectively. For SAI, just T-tests could be performed for test matches as only a single batsman using a SBBT had scored in ODI matches and the means for this group could be calculated. A one-way analysis of variance (ANOVA) was also calculated for highest scores, career averages and strike rates for first-class cricketers and List A statistics. All analyses were performed using R^[13]^ at a significance level of α = 0.05.

## Results

**Fig. 4:**
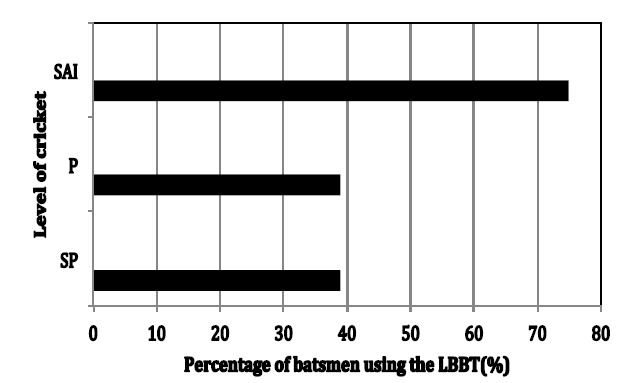
The percentage of batsmen using the LBBT among SP, PP and SAI cricketers (n = 155). PP, professional players; SP, semi-professional players; SAI, South African Internationals

In this study, 37% of SP and 38% of PP used a LBBT respectively (p>0.05) (Tables 1 and 2). Among the SP, 44% of players were classifier 1, 17% were classifier 2 and 37% were classifier 3 (LBBT). Among the PP, 34% of players were classifier 1, 26% were classifier 2 and 38% were classifier 3 (LBBT). There were also 75% of SAI (Table 3 and 4) who used the LBBT. In this study, the majority (75%) of SAI batsmen (playing at international level) used a LBBT, while only between 37% – 38% of batsmen on the other levels used the LBBT (Table 5 and Fig. 4). The percentage of cricketers using the LBBT is significantly different to those using the SBBT across the different levels (χ^2^ = 39.02, df =3, p = 0.001).

### Highest scores

The analysis was significant for both first class cricket, *F* (2, 114) = 19.369, *p* = .000 and List A cricket, *F* (2, 11) = 18.85, *p* = .000 (Table 6). Comparisons indicated that the high scores of cricketers at the amateur level was significantly different from the franchise level for both first-class cricket, *t*(103) = −3.18, *p* = .000 and List A cricket, *t*(102) = −3.61, *p* = .000. The high scores of cricketers at the amateur level was significantly different from the international level for both first class, *t*(64) = −6.32, *p* = .000 and List A cricket, *t*(27) = −8.21, *p* = .000. Similarly, high scores of cricketers at the franchise level was significantly different from the international level for both first class cricket, *t*(61) = 3.87, *p* = .000 and List A cricket, *t*(28) = −4.88, *p* = .000 (Table 6). When comparing cricketers in the PP group who either had a SBBT or a LBBT, the analysis was significant for highest scores in List A cricket (*t* = −2.02*; p* = 0.02) (Table 7).

### Career averages

The analysis was significant for both first-class cricket *F*(2, 114) = 10.89, *p* = .000 and List A cricket, *F*(2, 11) = 14.31, *p* = .000 (Table 6). Comparisons indicated that the averages of cricketers at the amateur level were significantly different from the franchise level for both first-class cricket, *t*(103) = 1.78, *p* = .038 and List A cricket, *t*(103) = 2.64, *p* = .005. The averages of cricketers at the amateur level were significantly different from the international level for both first-class cricket, *t*(36) = −7.75, *p* = .000 and List A cricket, *t*(31) = −7.75, *p* = .000. Similarly, career averages of cricketers at the franchise level were significantly different from the international level for both first-class cricket, *t*(46) = −5.93, *p* = .000 and List A cricket, *t*(31) = −5.22, *p* = .000 (Table 6).

When comparing cricketers in the SP group who either had a SBBT or a LBBT, the analysis was significant for career averages in first-class cricket (*t* = −2.19; *p* = 0.02) (Table 7). When comparing cricketers in the SP group (with a SBBT) to the PP group (with a LBBT), the analysis was significant for first-class cricket among highest scores (*t* = −3.01; *p* = 0.002) and career averages (*t* = −3.13*; p* = 0.001), and for List A cricket among highest scores (*t* = −3.94; *p* = 0.001) and career averages (*t* = −3.13; *p* = 0.001) (Table 7).

### Strike rates

As expected, there were no significant differences for all analyses on strike rates.

For test matches, the average highest score for South African International batsmen using a LBBT was significantly higher than that for batsmen using a SBBT (t = 2.34, p = 0.03) (Table 8). However, the use of a LBBT or a SBBT had no significant effect on total career runs (t = 1.70, p = 0.079), average run rate (t = 1.81, p = 0.056) and strike rate (t = 0.24, p = 0.41).

## Discussion

The main finding of this study has shown that a LBBT is commonly used by batsmen at the highest levels of cricket. Despite the small differences in percentages of SP and PP using the LBBT, this finding is nevertheless compatible with the interpretation in this study that the LBBT is more common at the highest levels of cricket (Fig. 4).

### Career averages and highest scores

There were also noticeably higher differences in the highest scores and career averages between all groups of players, as well as batsmen who either use a SBBT or a LBBT. As such, the LBBT acts as a selective factor among proficient batters at the highest levels of cricket batting.

This study has shown that the analysis of high scores and career averages was significant for both first-class cricket and List A cricket. Comparisons indicated that the high scores and career averages of cricketers at the amateur level were significantly different from the franchise level for first-class cricket and List A cricket. The high scores and career averages of cricketers at the amateur level were significantly different from the international level for both first-class and List A cricket. Similarly, high scores and career averages of cricketers at the franchise level were significantly different from the international level for first-class cricket and List A cricket.

This shows how performances (in the form of career averages and highest scores) increases with players who use the LBBT at the higher levels of the game in both limited overs and four-day cricket.

Wagon wheels were only available for the SAI groups, and therefore scoring areas and career statistics of these players will be clarified.

### Scoring areas

The SAI batsmen’s wagon wheels show interesting findings. Batsmen with a LBBT were found to score runs in more areas around the cricket field and in front of the wicket. By contrast, batsmen with a SBBT scored runs in selected areas around the cricket field only and roughly in front of the wicket (mostly behind square with shots such as the late cut and leg glance). Although the late cut to third man and leg glance to fine leg can be rewarding for batsmen, these are not shots that are always commonly played by batsmen within an innings. Some batsmen who are more defensive in their approach would score runs in selected areas around the cricket field whereas more aggressive batsmen may score runs in various parts of the cricket field. Interestingly, wagon wheel examples of three left-handed SAI batsmen with a LBBT (Quinton de Kock, Rilee Rossouw and David Miller) (Supplementary Figure 5) show that most of their runs are scored in front of the wicket and not behind square. All of their productive shots were on the on-side (the pull and on-drive). It is important to note that although there are multiple factors associated with scoring areas of batsmen (such as bowler’s lines and lengths, the grip of the batsman and formats of the game), backlift types of a batsman is not a single causative factor but rather one of the likely contributing factors among the many factors that contribute towards successful batting.

In addition to the SAI batsmen’s scoring areas, it is also worth noting their productive shots used during their highest scoring innings in either a test match or ODI. Batsmen with a LBBT (n = 9) had a leg glance and pull as their most productive shot (the leg-side), whereas batsmen with a SBBT (n = 3) had a cover drive as their most productive shot (off-side). From this, it is suggested that batsmen with a LBBT are more likely to go at a ball harder in the high scoring zone (on the leg-side) as opposed to a less high scoring zone (on the off-side). In this instance, batsmen who had a LBBT mostly used the leg glance and pull (leg-side), implying that these are not straight bat shots whereas batsmen with a SBBT mostly played a cover drive, implying a straight bat shot.

It is also important to consider the varied formats of the game. Batsmen would be more aggressive in one-day games as opposed to test matches. Further research is required in this area if there are variances in the batting backlift techniques of the same batsmen in all three formats of the game (tests, ODIs and Twenty20).

### Career statistics

Apart from Farhaan Behardien, three SAI batsmen with a SBBT were found to have the lowest high scores. Although an individual high score may not be an indicator for success, the observed pattern does support the idea that batsmen with a LBBT might be able to score runs more rapidly than batsmen with a SBBT. Furthermore, these three batsmen are part of the five batsmen from the SAI cohort that have the lowest strike rate in either tests or ODIs. Although the strike rate statistic is more pertinent in the ODI format of the game, it still raises the question of whether batsmen with a LBBT are able to score runs more rapidly than batsmen with a SBBT.

### The link between the LBBT and potential long-term success in cricket batting

The use of the LBBT may have decreased among a number of players as a result of exposure to traditional coaching methods and philosophies earlier in their careers.^[12]^ It has also been shown that coaching with the SBBT may be detrimental to cricketers’ future prospects^[12]^, as this study has shown that the LBBT produces better performances at the higher levels. In addition, if such players are not coached traditionally, they automatically hit the ball using the LBBT.

### Strengths and limitations

The strengths of this study are as follows:

- The ability to retrieve both completed batting records and video footage of all batsmen in this study.
- The sample number of 130 batsmen (SP: n = 69; PP: n = 49 and SAI: n = 12) is reflective of over half of the population sample of semi-professional and professional cricketers in South Africa.
- The analysis of all the six South African franchise teams (n = 49 batsmen) and nine out of the 13 South African semi-professional teams (n = 69).
- Each group of participants played in the same environment and in the same month, which limited a seasonal effect.
- Biomechanical and video analyses of players was also obtained objectively and was not self-reported.

With regards to the limitations:

- Only wagon wheels of the SAI could be sourced. However, this sample was sufficient to correlate the runs scored on the field with the players’ BBT.
- The dots per inch (dpi) quality for some of the videos with the SP and PP appeared to be inconsistent due to the variances in weather when testing, as well as varied camera distances behind the bowler.
- The researchers accounted for perspective error by limiting the type of videos observed and including horizontal lines in the background.
- Exact angles were not measured, while it was only possible to measure and report on angle ranges. This was because the analysis was conducted in a field setting (due to the number of players studied) and not in a laboratory.

### Coaching implications

All batsmen are unique in their technique and approach and will display attributes that are distinctive and suit them best as individual players. As scientists and coaches, the above should be taken into consideration in order to assist players with subtle discrepancies that may hinder their performance. Innovative coaching tools (specifically for the backlift), in the form of a coaching cricket bat and a mobile application, are also available for coaches and players to improve and assist with the coaching of the LBBT.^[14,15]^ A LBBT may not come naturally to some professional players. Coaches should also pay attention to the direction of the backlift with players, especially when correlating the backlift to various scoring areas on the cricket field. At semi-professional and professional levels, a coach can only do so much to ensure optimal performance and subtle technical optimisations.

## Conclusion

This study found that a LBBT is more common at the highest levels of cricket batsmanship. Batsmen at the various levels of cricket had percentages of the LBBT as follows: SP = 37%; PP = 38%; SAI = 75%; p = 0.001. The LBBT is a contributing factor for cricket players wanting to play cricket at the highest levels of the game. This study showed that there was also noticeable difference in the highest scores and career averages between all groups of players in general as well as batsmen who either use a SBBT or a LBBT. Cricket coaches need to pay attention to the direction of the backlift with players, especially when correlating the backlift to various scoring areas on the cricket field. Further in-depth research is required to fully investigate the change in batting backlift techniques among cricket players over a long-term period.

## Supplementary Information



## Figures and Tables

**Table 1:** A summary of the BBT characteristics at semi-professional level (SP) (n = 69)

Amateur team	N	Lateral BBT	Straight BBT	Classifier 1	Classifier 2	Classifier 3
Free State	3	1	2	2	0	1
North West	4	2	2	2	0	2
Gauteng	10	4	6	3	3	4
Easterns	10	3	7	5	2	3
Northerns	8	3	5	3	2	3
KwaZulu-Natal Inland	7	3	4	3	1	3
Border	7	3	4	3	1	3
Eastern Province	10	5	5	3	2	5
Western Province	10	2	8	7	1	2
**Total (%)**	**69**	**26 (37.7)**	**43 (62.3)**	**31 (44.9)**	**12 (17.4)**	**26 (37.7)**

BBT, batting backlift technique; N, sample number

**Table 2:** A summary of the BBT characteristics at professional level (PP) (n = 49)

Franchise team	N	Lateral BBT	Straight BBT	Classifier 1	Classifier 2	Classifier 3
Knights	8	4	4	2	2	4
Lions	5	1	4	3	1	1
Titans	8	4	4	2	2	4
Dolphins	10	5	5	3	2	5
Warriors	10	3	7	4	3	3
Cobras	8	2	6	3	3	2
**Total (%)**	**49**	**19 (38.8)**	**30 (61.2)**	**17 (34.7)**	**13 (26.5)**	**19 (38.8)**

BBT, batting backlift technique; N, sample number

**Table 3:** Characteristics and performances of the South African National Team up to and including the 2015/2016 season at Test and ODI levels.

Player	Runs Tests	Runs ODI	Test			ODI			Classifier	BBT

			High score	Average	Strike rate	High score	Average	Strike rate		
					
AB de Villiers	8074	8621	**278**	50.5	53.7	162*	54. 6	100.2	3	Lateral
Hashim Amla	7358	6204	**311** *	51.5	50.1	159	52.1	89.1	3	Lateral
Quinton de Kock	407	2319	**129** *	45.2	66.2	138*	42.9	91.7	3	Lateral
Dean Elgar	1249	98	**121**	36.7	45.2	42	24.5	61.3	1	Straight
Faf du Plessis	1682	2944	**137**	41.0	39.7	133*	39.8	85.7	3	Lateral
JP Duminy	1423	4028	**166**	32.3	42.8	150*	38.7	83.3	3	Lateral
Temba Bavuma	383	-	**102**	38.3	47.2	-	-	-	1	Straight
Stephen Cook	140	-	**115**	70.0	48.8	-	-	-	3	Lateral
Rilee Rossouw	-	860	-	-	-	**132**	33.1	94.6	3	Lateral
David Miller	-	1819	-	-	-	**138** *	35.0	100.0	3	Lateral
Farhaan Behardien	-	767	-	-	-	**70**	30.7	97.3	3	Lateral
Stiaan van Zyl	355	-	**101** *	27.3	54.0	-	-	-	2	Straight

BBT, batting backlift technique; ODI, one day internationals.

* indicates not out; – indicates player did not play Tests/ODI format.

Player’s highest score is in bold text. These stats were accessed during June 2016.

**Table 4:** Performances of the South African National Team batsmen up to and including the 2015/2016 season at First-Class and List A levels

Player	Runs First-Class Total	Runs List A Total	First-Class			List A			Classifier	BBT

			High score	Average	Strike rate	High score	Average	Strike rate		
					
AB de Villiers	9961	10721	278*	49.8	55.5	162*	54.1	-	3	Lateral
Hashim Amla	15477	8562	311*	49.7	-	159	45.3	-	3	Lateral
Quinton de Kock	3225	4555	194	48.8	79.9	178	41.0	95.9	3	Lateral
Dean Elgar	9858	4223	268	43.8	49.3	117	39.4	77.1	1	Straight
Faf du Plessis	6763	7858	176	40.2	-	185	45.1	88.9	3	Lateral
JP Duminy	6699	6228	260*	47.1	49.9	150*	37.7	81.6	3	Lateral
Temba Bavuma	5554	1607	162	37.7	50.6	113	29.2	80.9	1	Straight
Stephen Cook	12983	5182	390	40.5	-	127*	38.9	78.3	3	Lateral
Rilee Rossouw	5940	4668	319	44.3	63.6	137	38.5	93.5	3	Lateral
David Miller	2851	4595	177	35.6	56.8	138*	40.3	101.7	3	Lateral
Farhaan Behardien	5403	4070	150*	39.7	54.0	113*	37.3	94.4	3	Lateral
Stiaan van Zyl	8401	3131	172	42.8	51.5	114*	35.9	73.7	1	Straight

BBT, batting backlift technique; ODI, one day internationals.

* indicates not out; – indicates player did not play Tests/ODI format.

These stats were accessed during March 2017.

**Table 5:** Percentage of players across different professional levels applying the LBBT or SBBT, assigned to classifiers 1 – 3

Level	N	Backlift batting technique (%)		Classifier (%)		

		LBBT	SBBT	1	2	3
		
Semi-professional (SP)	69	38	62	45	17	38
Professional (PP)	49	39	61	35	26	39
South African International (SAI)	12	75	25	17	8	75
**Total**	**155**	**51**	**49**	**32**	**17**	**51**

LBBT, lateral batting backlift technique; SBBT, straight batting backlift technique; N, sample number

**Table 6:** First-Class and List A performances for SP, PP and SAI groups

Level	N	First-Class			List A		

		High score	Average	Strike rate	High score	Average	Strike rate
		
Semi-professional (SP)	69	110	27.0	51.5	56	22.4	74.6
Professional (PP)	49	146	30.4	50.5	91	28.0	77.4
South African International (SAI)	12	232	42.1	45.5	146	38.3	86.6

N, sample number. High score refers to overall average highest score made by all the players in the particular match format. Average refers to the overall average runs scored by all of the players in the particular match format. Strike rate refers to the overall strike rate achieved by all of the players in the particular match format.

**Table 7:** First-Class and List A performances for SP and PP groups separated into a SBBT or a LBBT

Level	N	First-Class			List A		

		High score	Average	Strike rate	High score	Average	Strike rate
		
Amateur SBBT	62	111	24.7	51.6	52	20,8	75.4
Amateur LBBT	38	107	29.7	51.3	62	24,3	73.6
Franchise SBBT	61	138	28.7	48.9	84	27,1	75.3
Franchise LBBT	39	164	33.3	53.2	104	29,4	80.8

SP, Semi-professional; PP, Professional; LBBT, lateral batting backlift technique; SBBT, straight batting backlift technique; N, sample number. High score refers to overall average highest score made by all the players in the particular match format. Average refers to the overall average runs scored by all of the players in the particular match format. Strike rate refers to the overall strike rate achieved by all of the players in the particular match format.

**Table 8:** Mean performance per cricketer using lateral (LBBT) or straight (SBBT) backlift batting techniques at South African International (SAI) level

BBT	N	Test				ODI			

		Total runs	Average runs	Strike rate	High score	Total runs	Average runs	Strike rate	High score
		
LBBT	9	3181 ± 1189	48.4 ± 4.2	50.2 ± 3.1	174 ± 24	3445 ± 915	40.9 ± 2.8	92.7 ± 2.1	120 ± 15
SBBT	3	662 ± 293	34.1 ± 3.4	48.8 ± 2.6	112 ± 8	98 ± 0.0	24.5 ± 0	61.3 ± 0	42 ± 0.0
**Total**	**12**	**2341 ± 895**	**43.6 ± 3.6**	**49.7 ± 2.2**	**153 ± 19**	**3073 ± 808**	**39.0 ± 2.8**	**89.2 ± 3.4**	**101 ± 16**

Data expressed as Mean ± Standard Error

BBT, batting backlift technique; LBBT, lateral batting backlift technique; SBBT, straight batting backlift technique; N, sample number; ODI, one day internationals
