# Supplementary material for: The lateral batting backlift technique: is it a contributing factor to success for professional cricket players at the highest level?
Source: S Afr J Sports Med. 2019 Jan 1;31(1):v31i1a5460. doi: 10.17159/2078-516X/2019/v31i1a5460 (PMC9924531; doi:10.17159/2078-516X/2019/v31i1a5460)
Supplement: Supplementary file 1 [file 2078-516X-31-v31i1a5460-s001.pdf]

Supplementary Table 9: Characteristics and performances of the Free State cricket team (n = 3)

Player	Mat	Inn	NO	Runs	First Class			List A			Classifier	BBTT
					High Score	Average	Strike Rate	High Score	Average	Strike Rate		
T. Ntuli	10	13	6	35	**9**	5.0	31.2	4*	-	77.7	1	Straight
S. Arends	2	3	0	1	**1**	0.3	6.6	-	-	-	1	Straight
J. L. du Plooy	15	24	2	816	**181**	37.0	51.9	85*	154.5	89.0	3	Lateral

*BBTT = Batting Backlift Technique Type; Inn = Innings; Mat = Matches; NO = Not Outs; * = Not out; - = did not play Tests/ODI; Highest scores = BOLD*

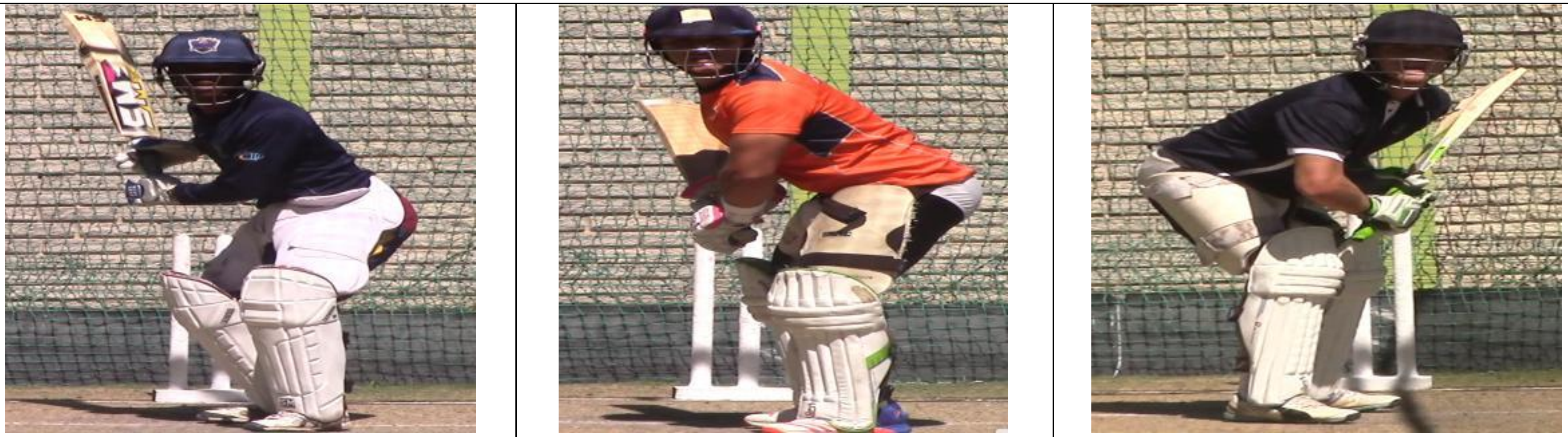

T. Ntuli (S; HS = 9)	S. Arends (S; HS = 1)	J.L. du Plooy (L; HS = 181)

Supplementary Figure 5.1: Batting backlift technique type of Free State cricket players (n = 3)

S = SBBT; L = LBBT; HS = Highest Score

Supplementary Table 10: Characteristics and performances of the Knights cricket team (n = 8)

Player	Mat	Inn	NO	Runs	First Class			List A			Classifier	BBTT
					High Score	Average	Strike Rate	High Score	Average	Strike Rate		
M. N. Erlank	88	142	13	3482	**163***	26.9	43.0	72	22.6	73.3	3	Lateral
P. J. van Biljon	72	121	14	4264	**215***	39.8	52.5	134*	32.6	82.4	3	Lateral
R. R. Hendricks	105	185	12	5684	157	32.8	51.6	**181**	36.6	83.4	2	Straight
L. L. L. Sesele	65	116	5	2414	**177**	21.7	49.7	56	17.5	65.5	2	Straight
P. Botha	71	109	17	3359	**109***	36.5	59.7	76	23.3	83.4	3	Lateral
R.S. Second	70	118	9	4224	**210**	38.7	48.4	135*	49.1	74.1	1	Straight
W. L. Coetsee	118	198	23	5598	**202***	31.9	57.9	130*	25.2	88.9	2	Straight
T. M. Bodibe	121	214	16	5074	**148**	25.6	47.6	88	19.4	62.8	3	Lateral

*BBTT = Batting Backlift Technique Type; Inn = Innings; Mat = Matches; NO = Not Outs; * = Not out; Highest scores = BOLD*

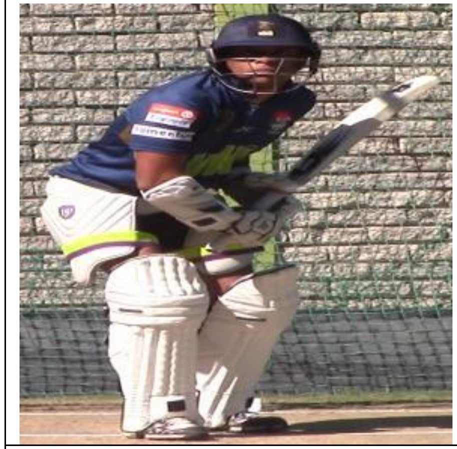	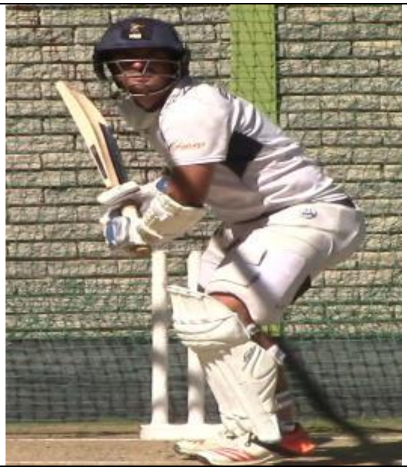	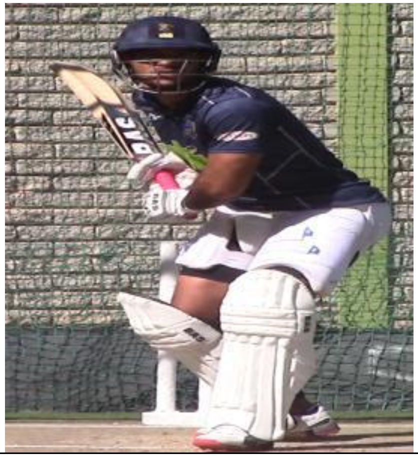	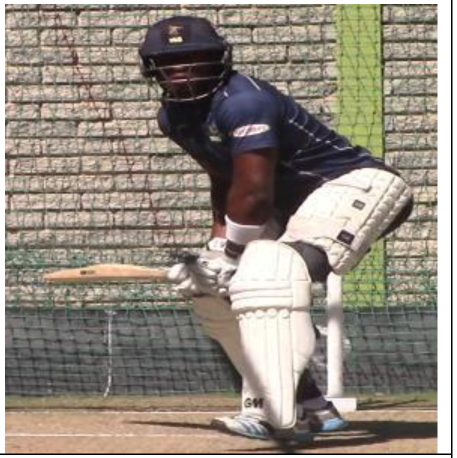
M.N. Erlank (L; HS = 163*)	P.J. van Biljon (L; HS = 215*)	R.R. Hendricks (S; HS = 181)	T.M. Bodibe (L; HS = 148)
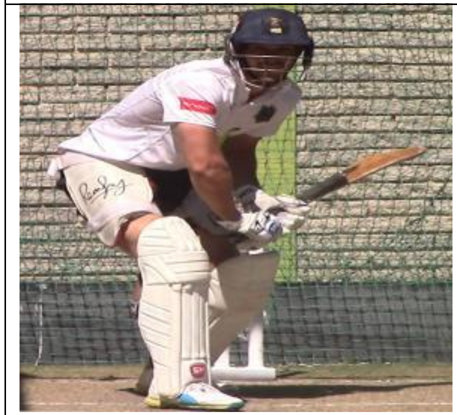	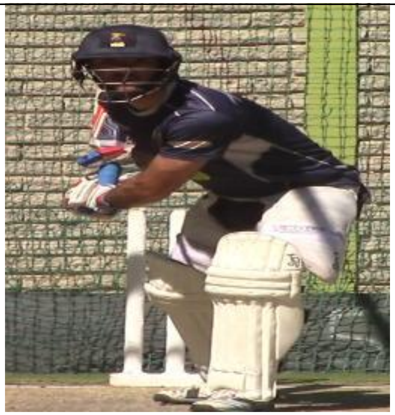	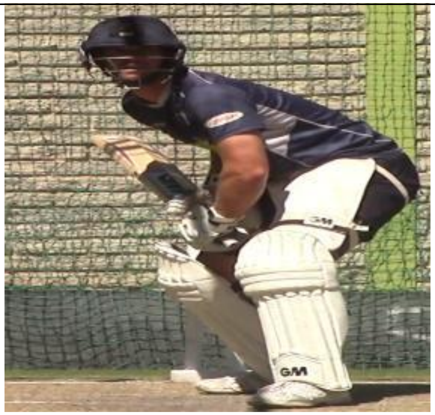	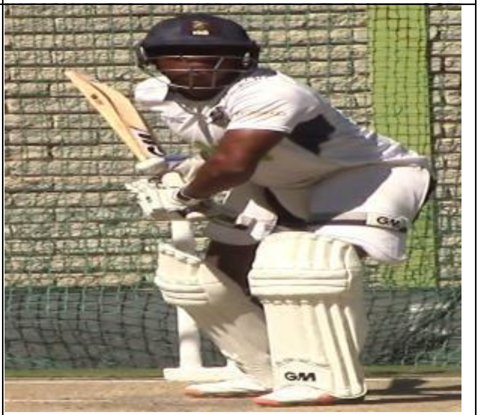
P. Botha (L; HS = 109*)	R.S. Second (S; HS = 210)	W.L. Coetzee (S; HS = 202*)	L.L.L. Sesele (S; HS = 177)

Supplementary Figure 5.2: Batting backlift technique type of the Knights players (n = 8)

S = SBBT; L = LBBT; HS = Highest Score

Supplementary Table 11: Characteristics and performances of the Easterns cricket team (n = 10)

Player	Mat	Inn	NO	Runs	First Class			List A			Classifier	BBTT
					High Score	Average	Strike Rate	High Score	Average	Strike Rate		
J. Snyman (L)	14	22	0	605	**150**	27.5	59.3	121*	41.8	74.4	2	Straight
D. Stanley (R)	53	74	31	496	**55**	11.5	39.1	42	21.5	76.7	1	Straight
K. Apea-Adu (R)	7	8	2	51	**31**	8.5	35.6	5*	-	66.6	1	Straight
C.J. Dala (R)	35	39	15	313	**79***	13.0	40.3	20	11.4	77.4	1	Straight
E. H. Kemm (L)	32	55	4	1829	**136**	35.8	37.1	81	32.0	62.0	3	Lateral
W. B. Marshall (R)	17	31	0	860	**103**	27.7	60.6	89	37.5	99.1	1	Straight
T. A. Bula (R)	95	157	23	3909	**106**	29.1	61.7	91	28.0	85.9	3	Lateral
W. Coulentianos (L)	34	57	5	1867	**171**	35.9	53.8	55	43.4	66.3	1	Straight
E. R. Links (R)	50	72	16	1323	**101***	23.6	52.2	42	16.7	64.4	3	Lateral
V. P. Moore (R)	27	37	10	385	**48***	14.2	32.9	18*	13.0	70.2	2	Straight

*BBTT = Batting Backlift Technique Type; Inn = Innings; Mat = Matches; NO = Not Outs; * = Not out; - = did not play Tests/ODI; Highest scores = BOLD*

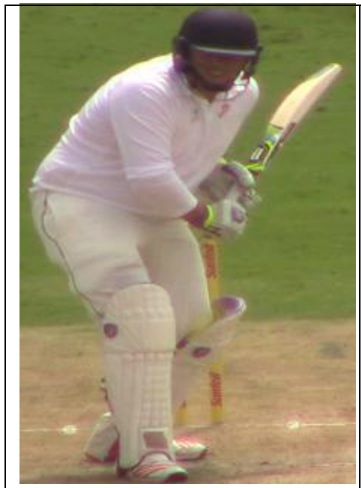	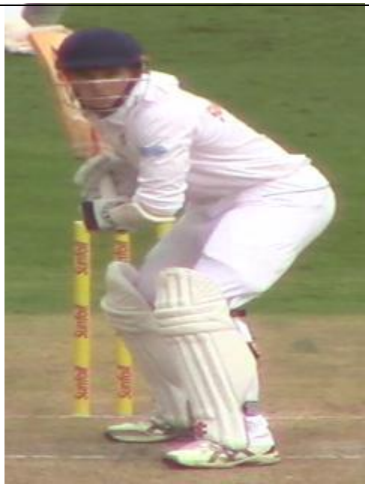	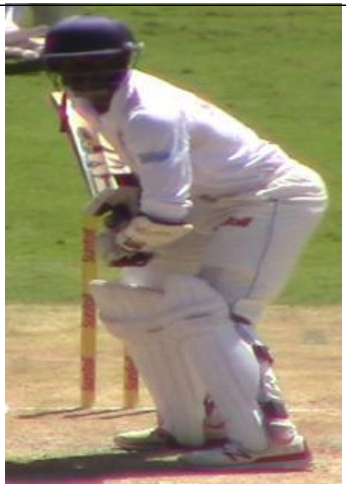	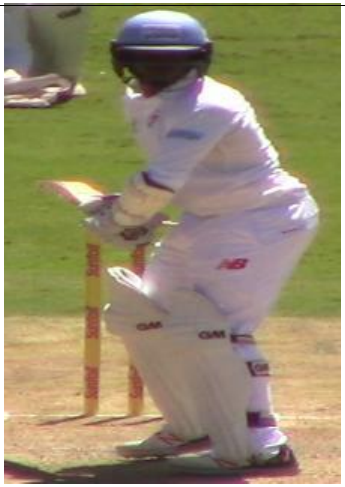	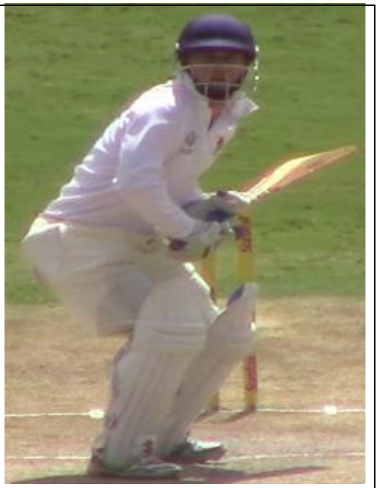
Snyman (S; HS = 150)	Stanley (S; HS =55)	Apea-Adu (S; HS = 31)	Dala (S; HS = 79*)	Kemm (L; HS = 136)
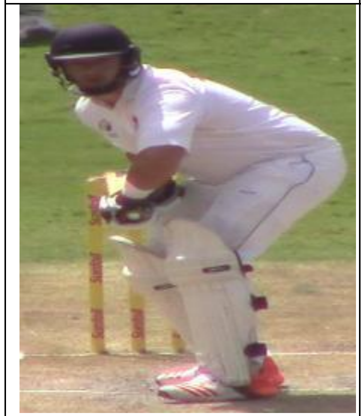	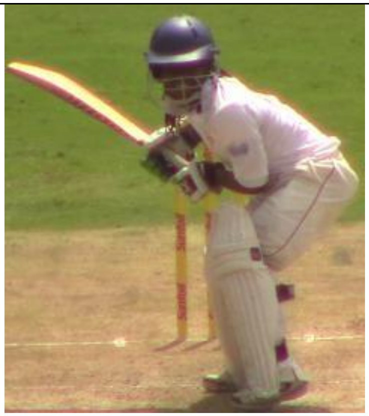	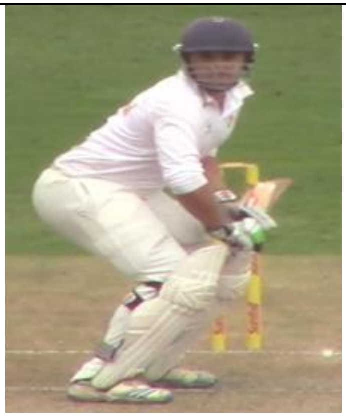	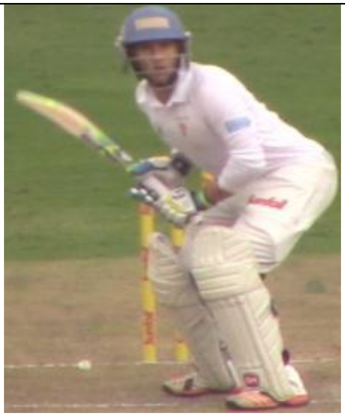	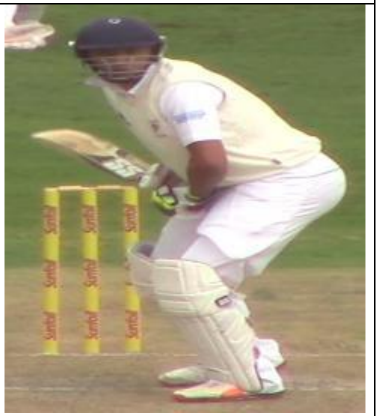
Marshall (S; HS = 103)	Bula (L; HS = 106)	Coulentianos (S; HS = 171)	Links (L; HS = 101*)	Moore (S; HS = 48*)

Supplementary Figure 5.3: Batting backlift technique type of the Easterns players (n = 10)

S = SBBT; L = LBBT; HS = Highest Score

Supplementary Table 12: Characteristics and performances of the Gauteng cricket team (n = 10)

Player	Mat	Inn	NO	Runs	First Class			List A			Classifier	BBTT
					High Score	Average	Strike Rate	High Score	Average	Strike Rate		
K. Mogotsi	10	18	2	358	**69**	22.3	47.9	61	21.1	59.9	3	Lateral
Y. Valli	21	36	7	790	**130***	27.2	41.1	31	17.8	54.3	3	Lateral
D. P. Conway	77	121	10	4189	142	37.7	54.9	**152**	44.1	82.7	3	Lateral
S. Pillay	28	45	9	1144	**138***	31.7	44.3	81	30.2	69.9	1	Straight
N.P. Mvelase	8	11	3	90	18	11.2	75.0	**25**	15.0	95.2	2	Straight
B. Dial	8	13	2	306	**94***	27.8	50.2	18	7.0	50.0	2	Straight
S. Jamison	31	33	8	446	**49**	17.8	58.3	15	11.6	62.5	2	Straight
D. Potgieter	4	7	1	99	39	16.5	55.6	**41**	36.5	85.8	2	Straight
G. Roelofsen	1	1	0	4	**-**	-	-	**4**	4.0	100.0	2	Straight
M. K. McGillivray	27	39	8	557	**69**	17.9	50.3	67	29.3	86.6	3	Lateral

*BBTT = Batting Backlift Technique Type; Inn = Innings; Mat = Matches; NO = Not Outs; * = Not out; - = did not play Tests/ODI; Highest scores = BOLD*

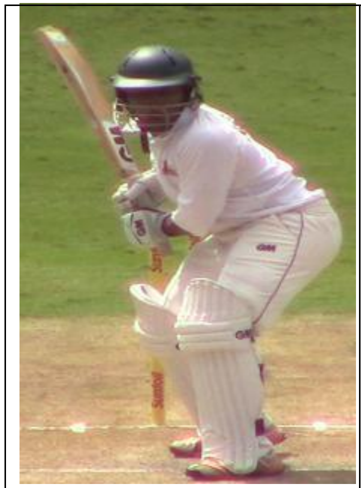	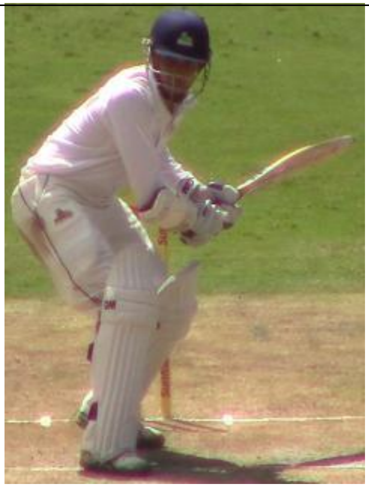	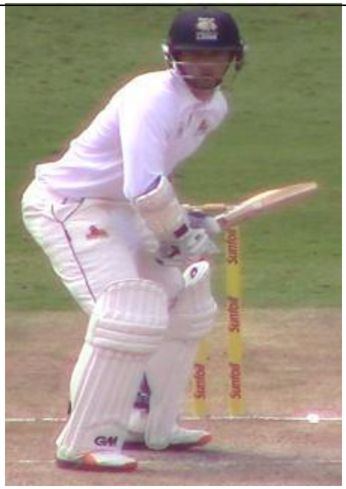	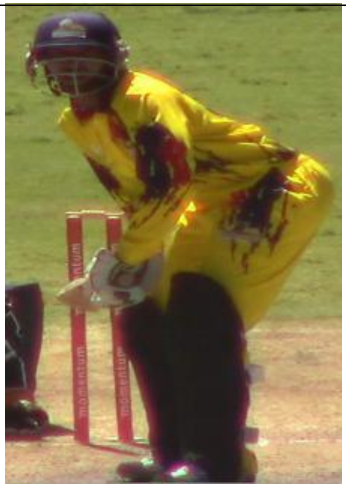	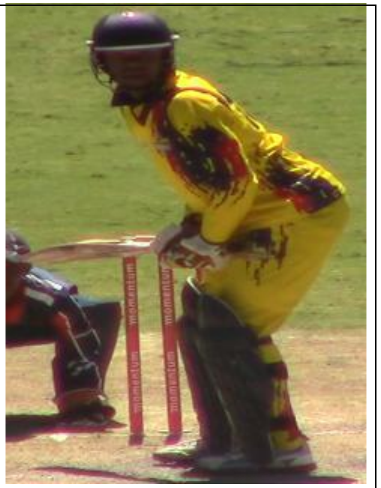
Mogotsi (L; HS = 69)	Valli (L; HS = 130*)	Conway (L; HS = 152)	Pillay (S; HS = 138*)	Mvelase (S; HS = 25)
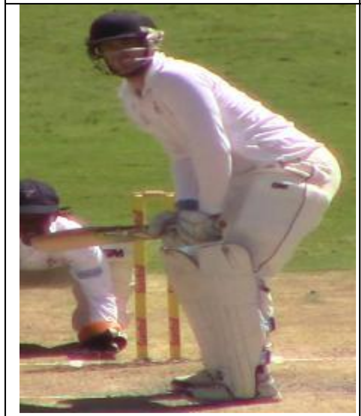	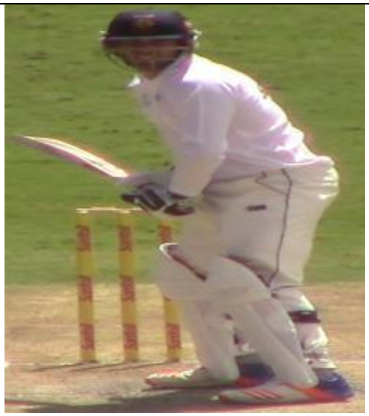	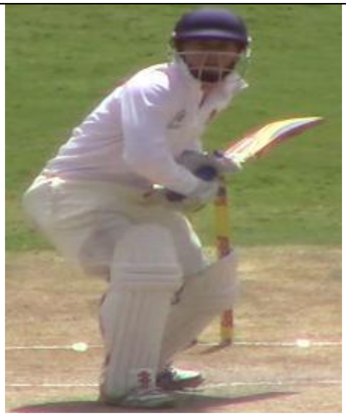	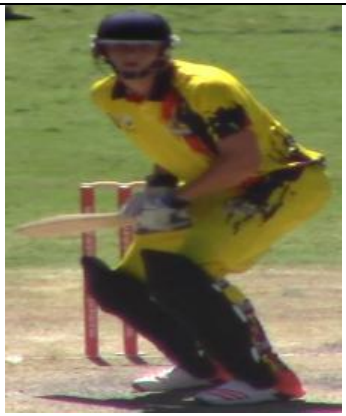	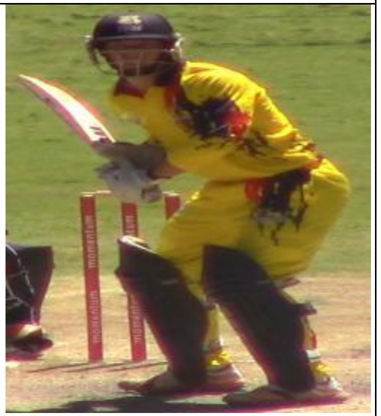
Dial (S; HS = 94*)	Jamison (S; HS = 49)	Potgieter (S; HS = 41)	Roelofsen (S; HS = 4)	McGillivray (L; HS = 69)

Supplementary Figure 5.4: Batting backlift technique type of the Gauteng players (n = 10)

S = SBBT; L = LBBT; HS = Highest Score

Supplementary Table 13: Characteristics and performances of the North West cricket team (n = 4)

Player	Mat	Inn	NO	Runs	First Class			List A			Classifier	BBTT
					High Score	Average	Strike Rate	High Score	Average	Strike Rate		
K. Rapulana	53	89	5	1930	**112**	22.9	50.4	56	19.1	65.2	1	Straight
M. J. Ackerman	4	7	0	77	31	11.0	48.4	**80**	31.5	86.3	2	Straight
F.J. Lubbe	4	5	1	254	**106**	63.5	51.5	14	7.6	92.0	3	Lateral
W.J. Lubbe	14	23	4	587	**93**	30.8	60.3	78	30.6	72.6	3	Lateral

*BBTT = Batting Backlift Technique Type; Inn = Innings; Mat = Matches; NO = Not Outs; * = Not out; Highest scores = BOLD*

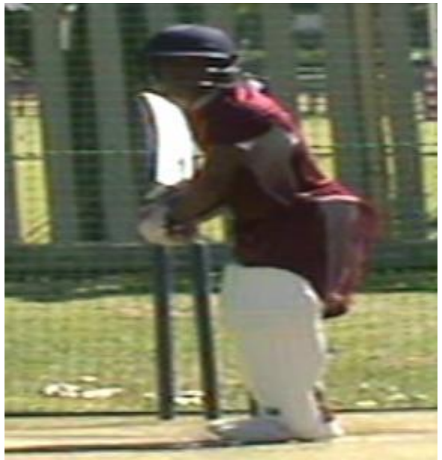	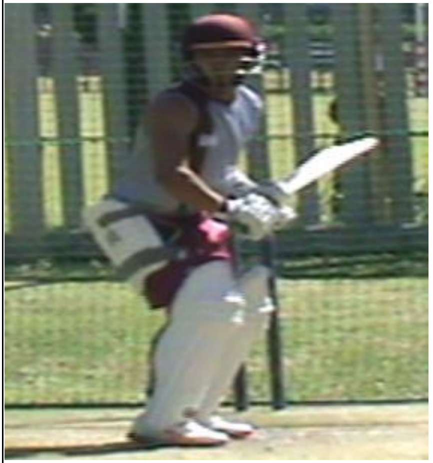	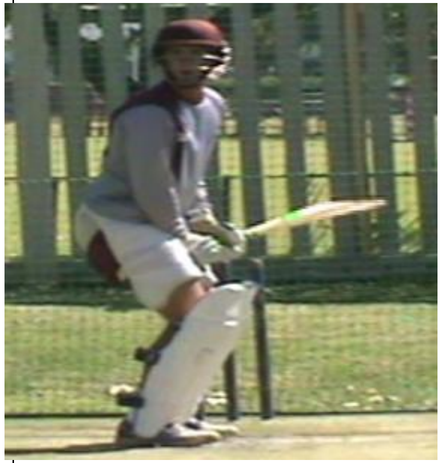	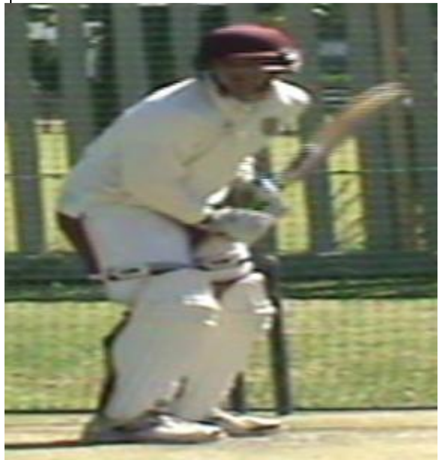
K. Rapulana (S: HS = 112)	M.J. Ackerman (S; HS = 80)	F.J. Lubbe (S; HS = 106)	W.J. Lubbe (S; HS = 93)

Supplementary Figure 5.5: Batting backlift technique type of the North West players (n = 4)

S = SBBT; L = LBBT; HS = Highest Score

Supplementary Table 14: Characteristics and performances of the Lions cricket players (n = 5)

Player	Mat	Inn	NO	Runs	First Class			List A			Classifier	BBTT
					High Score	Average	Strike Rate	High Score	Average	Strike Rate		
S. C. Cook	171	315	28	11890	**390**	41.4	48.7	127*	38.8	78.1	3	Lateral
H. E. van der Dussen	83	137	14	5046	**166**	41.0	46.2	134*	45.3	73.7	2	Straight
A. N. Petersen	216	371	19	14062	**286**	39.9	51.7	142*	36.2	82.6	1	Straight
T. Bavuma	90	146	21	4900	**162**	39.2	51.2	108*	26.7	80.0	1	Straight
D. Pretorius	32	44	5	1510	**177**	38.7	71.6	77*	41.0	98.4	2	Straight

*BBTT = Batting Backlift Technique Type; Inn = Innings; Mat = Matches; NO = Not Outs; * = Not out; Highest scores = BOLD*

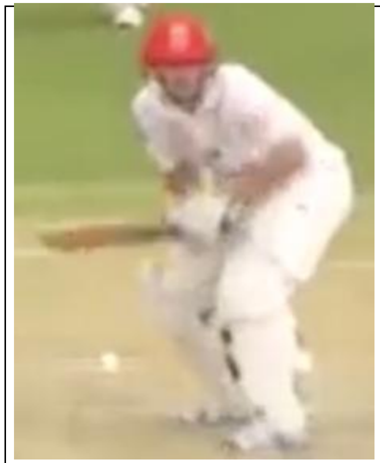	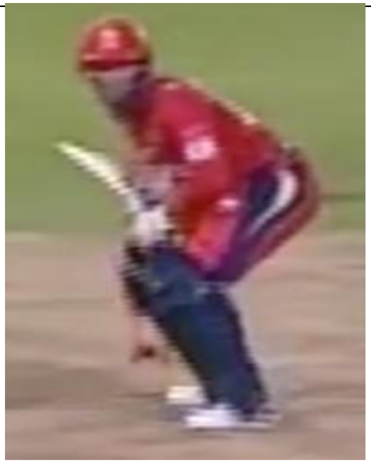	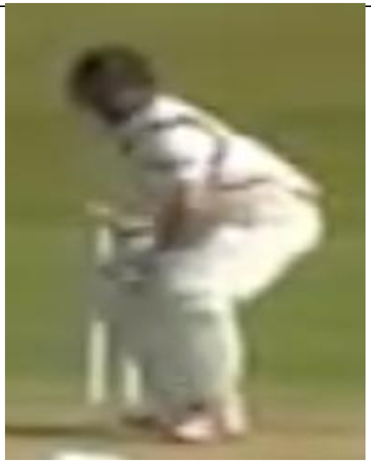	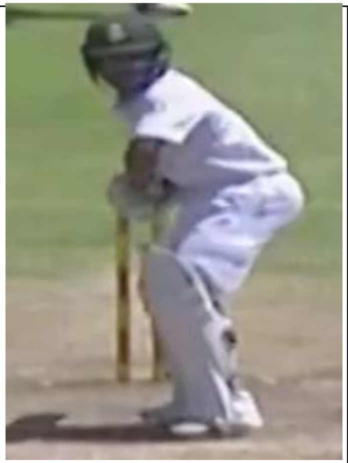	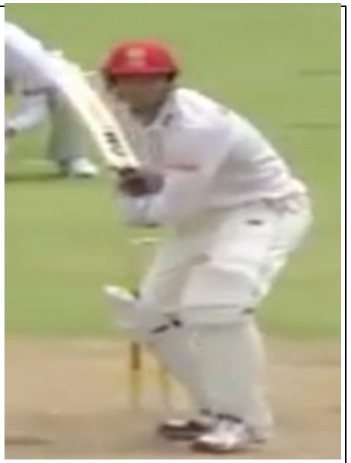
Cook (L; HS = 390)	v.d. Dussen (S; HS = 166)	Petersen (S; HS = 286)	Bavuma (S; HS = 162)	Pretorius (S; HS = 177)

Supplementary Figure 5.6: Batting backlift technique type of the Lions players (n = 5)

S = SBBT; L = LBBT; HS = Highest Score

Supplementary Table 15: Characteristics and performances of the Northerns cricket team (n = 8)

Player	Mat	Inn	NO	Runs	First Class			List A			Classifier	BBTT
					High Score	Average	Strike Rate	High Score	Average	Strike Rate		
J.J. Pienaar	86	142	13	4008	**185**	31.0	66.3	119	24.9	89.2	1	Straight
V.B. Mahlangu	16	24	1	366	**61**	15.9	46.0	16	10.0	48.7	2	Straight
G. L. van Buuren	54	82	14	3362	**235**	49.4	66.3	119*	31.0	84.8	1	Straight
A.K. Markram	20	32	2	1126	**182**	37.5	59.9	111	35.6	88.0	3	Lateral
S. Naidoo	57	84	7	1893	**119**	24.5	49.0	60	17.4	60.1	3	Lateral
H. Klaasen	42	63	12	2505	**201**	49.1	70.0	70*	22.4	80.5	2	Straight
S. von Berg	75	114	16	2738	**105***	27.9	55.5	47*	14.0	79.8	3	Lateral
S.A. Mothoa	9	17	8	97	**23***	10.7	44.2	-	-	-	2	Straight

*BBTT = Batting Backlift Technique Type; Inn = Innings; Mat = Matches; NO = Not Outs; * = Not out; - = did not play Tests/ODI; Highest scores = BOLD*

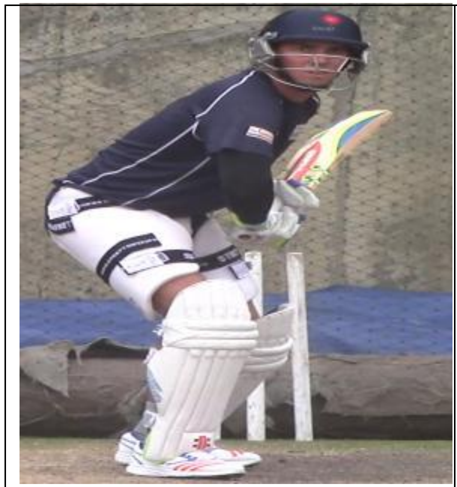	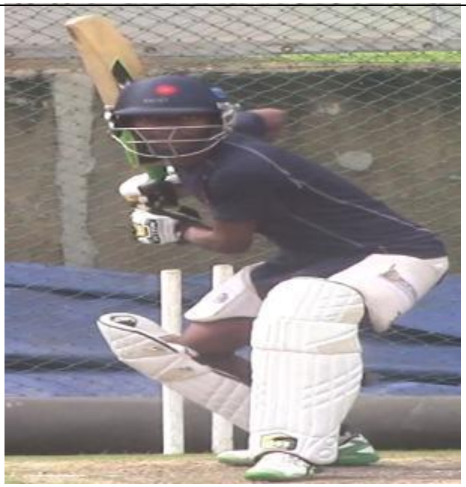	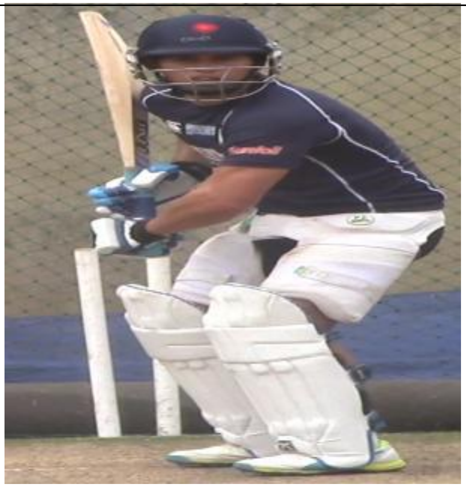	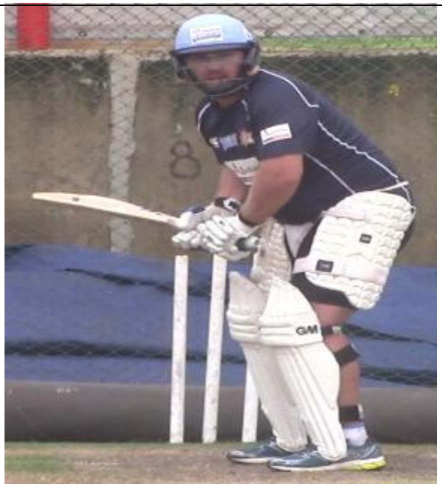
J.J. Pienaar (S; HS = 185)	V.B. Mahlangu (S; HS = 61)	G.L. van Buuren (S; HS = 235)	S. von Berg (L; HS =)
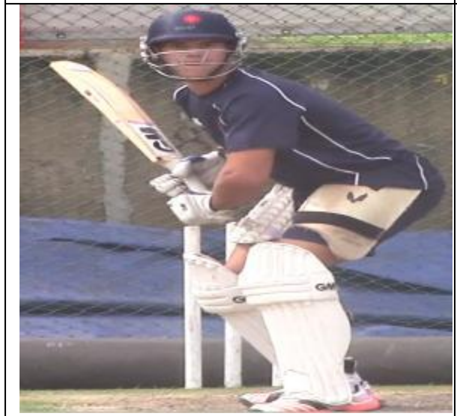	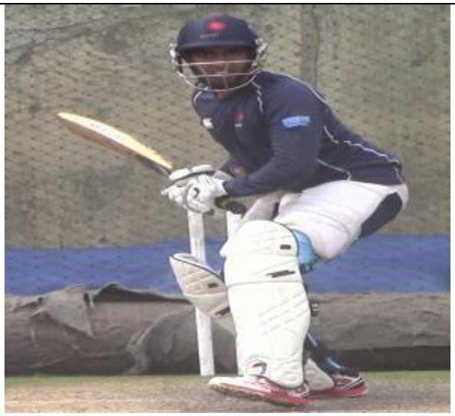	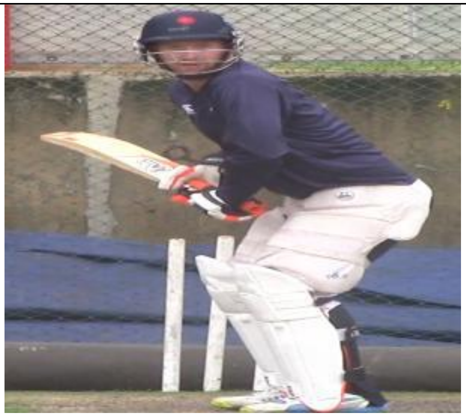	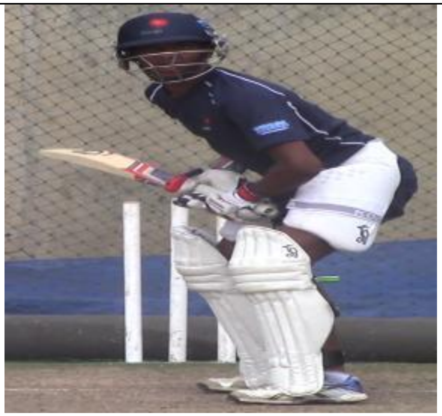
A.K. Markram (L; HS = 182)	S. Naidoo (L; HS = 119)	H. Klaasen (S; HS = 201)	S.A. Mothoa (S; HS = 23*)

Supplementary Figure 5.7: Batting backlift technique type of the Northerns players (n = 8)

S = SBBT; L = LBBT; HS = Highest Score

Supplementary Table 16: Characteristics and performances of the Titans cricket team (n = 8)

Player	Mat	Inn	NO	Runs	First Class			List A			Classifier	BBTT
					High Score	Average	Strike Rate	High Score	Average	Strike Rate		
H. G. Kuhn	121	211	21	8298	**244***	43.6	-	141*	30.8	85.1	3	Lateral
Q. de Kock	34	57	7	2404	**194**	48.0	81.3	138*	40.0	93.5	3	Lateral
D. Elgar	126	217	21	8751	**268**	44.6	49.9	117	39.5	77.1	1	Straight
T. G. Mokoena	89	150	7	4224	**217**	29.5	62.6	115*	17.4	75.3	2	Straight
T. B. de Bruyn	26	47	4	2009	**202***	46.7	65.9	152*	36.3	81.6	3	Lateral
H. Davids	123	122	10	6658	158	31.4	-	**166**	30.4	33.3	3	Lateral
M.Q. Adams	63	102	8	3773	**167**	40.1	59.8	121*	38.9	95.7	2	Straight
E. L. Hawken	21	23	1	252	**54**	11.4	35.7	18	12.5	69.4	2	Straight

*BBTT = Batting Backlift Technique Type; Inn = Innings; Mat = Matches; NO = Not Outs; * = Not out; - = did not play Tests/ODI; Highest scores = BOLD*

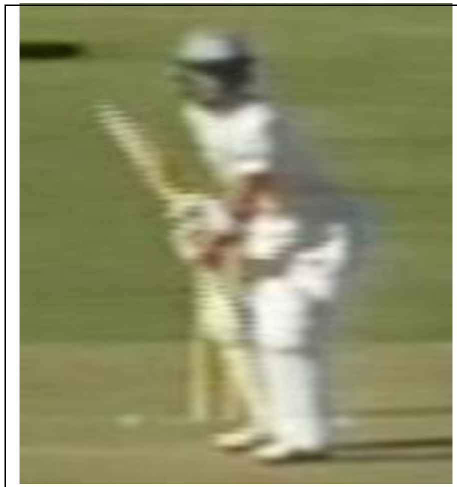	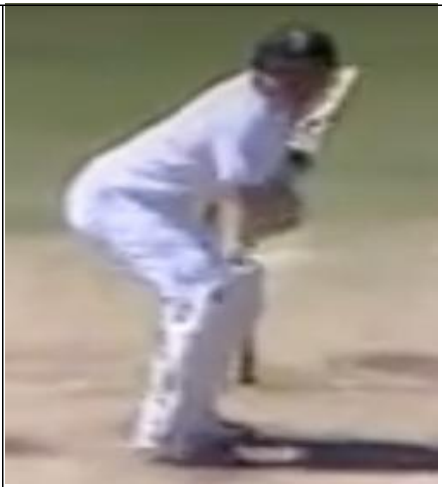	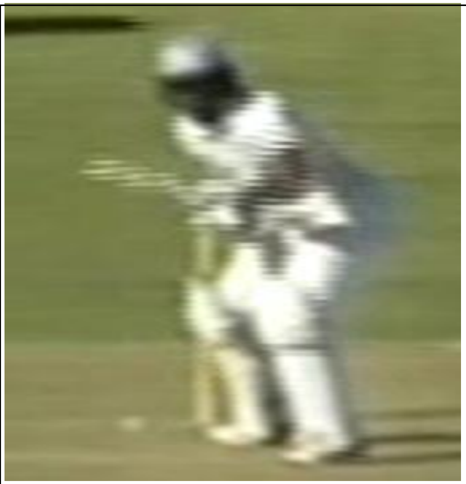	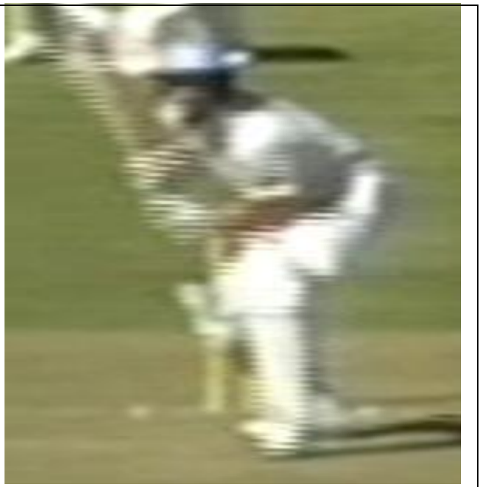
H.G. Kuhn (L; HS = 244*)	D. Elgar (S; HS = 268)	T.G. Mokoena (S; HS = 202*)	T.B. de Bruyn (L; HS = 217)
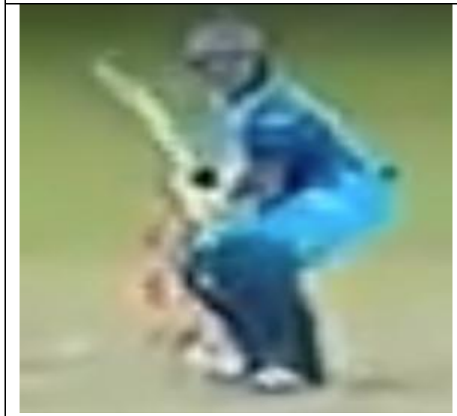	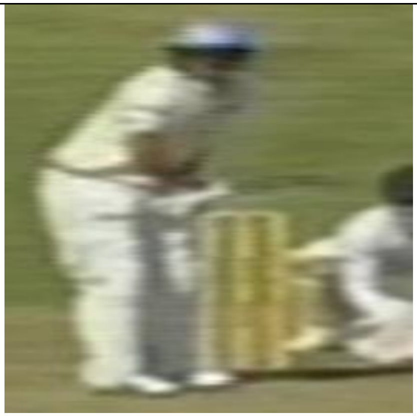	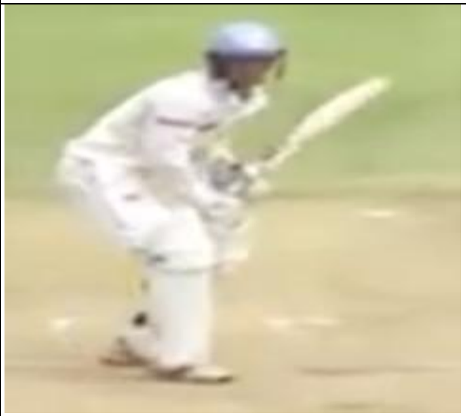	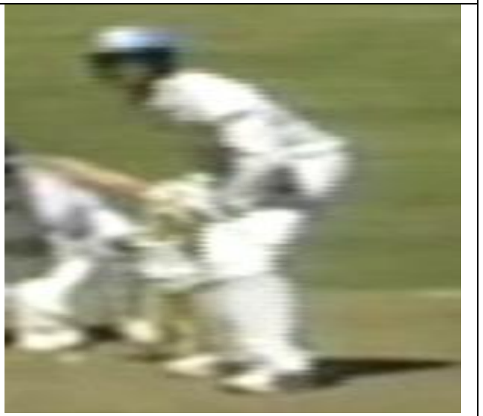
H. Davids (L; HS = 166)	M.Q. Adams (S; HS = 167)	Q. de Kock (L; HS = 194)	E.L. Hawken (S; HS = 54)

Supplementary Figure 5.8: Batting backlift technique type of the Titans players (n = 8)

S = SBBT; L = LBBT; HS = Highest Score

Supplementary Table 17: Characteristics and performances of the Kwa-Zulu Natal Inland cricket team (n = 7)

Player	Mat	Inn	NO	Runs	First Class			List A			Classifier	BBTT
					High Score	Average	Strike Rate	High Score	Average	Strike Rate		
D.J. van Wyk	124	212	8	8048	**178**	39.4	53.9	118	34.0	79.5	1	Straight
K. Nipper	77	125	19	3677	**151***	34.6	60.1	100*	27.9	85.6	3	Lateral
R. Pretorius	37	61	6	1319	66*	23.9	58.1	**71**	34.5	93.8	2	Straight
G. Dukes	12	24	3	415	**54**	19.7	58.0	47*	27.0	79.4	3	Lateral
L. Mosena	73	118	4	2612	**105**	22.9	48.2	73	23.3	60.4	3	Lateral
K. R. Kishun	18	23	6	368	**96**	21.6	38.4	37	12.1	59.5	1	Straight
G. Hume	68	89	23	1266	**105**	19.1	45.9	30	17.9	60.4	1	Straight

*BBTT = Batting Backlift Technique Type; Inn = Innings; Mat = Matches; NO = Not Outs; * = Not out; Highest scores = BOLD*

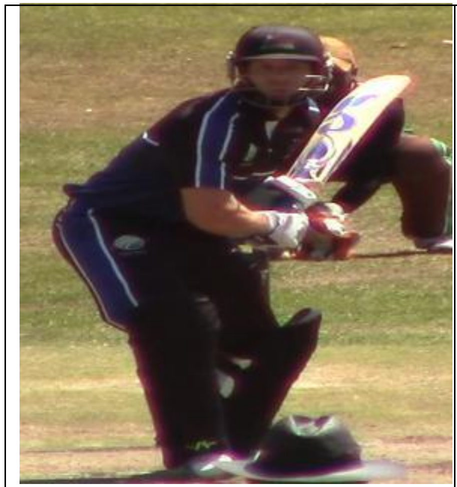	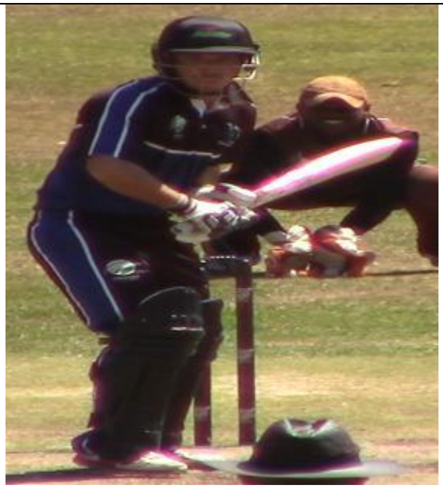	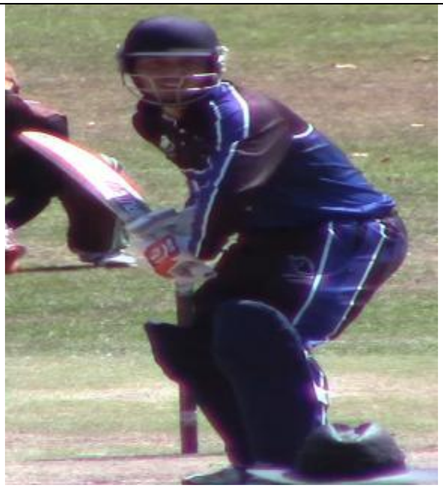	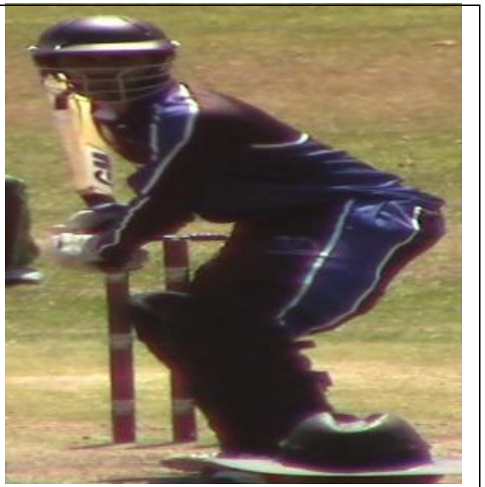
D.J. van Wyk (S; HS = 178)	K. Nipper (L; HS = 151*)	R. Pretorius (S; HS = 66*)	K.R. Kishun (S; HS = 96)
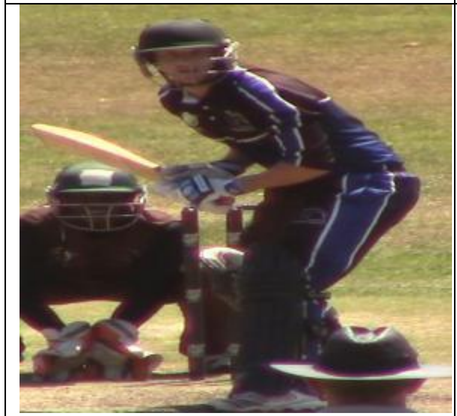	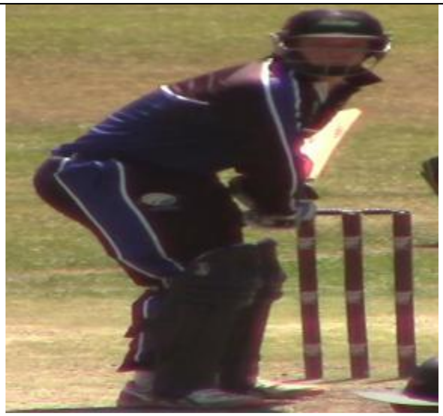	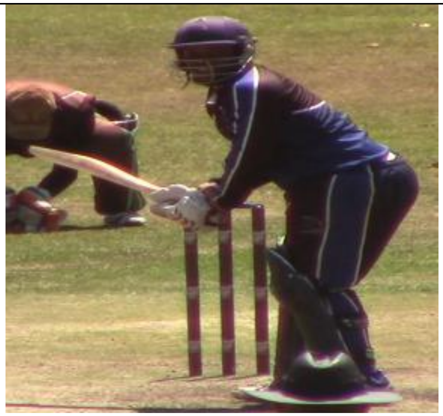	
G. Dukes (L; HS = 54)	G. Hume (S; HS = 105)	L. Mosena (L; HS = 105)	

Supplementary Figure 5.9: Batting backlift technique type of the Kwa-Zulu Natal Inland players (n = 7)

Supplementary Table 18: Characteristics and performances of the Dolphins cricket team (n = 10)

Player	Mat	Inn	NO	Runs	First Class			List A			Classifier	BBTT
					High Score	Average	Strike Rate	High Score	Average	Strike Rate		
K. Zondo	81	128	3	3503	**175**	28.0	48.0	110	28.6	75.5	1	Straight
V. B. van Jaarsveld	92	152	6	5698	**160**	39.0	63.0	118	37.2	86.6	3	Lateral
J. D. Vandiar	72	118	9	3598	**172***	36.3	59.7	130	31.2	87.3	3	Lateral
D. Smit	116	172	31	5086	**156***	36.0	46.8	109	31.6	76.8	3	Lateral
C. Alexander	90	103	39	802	**54**	12.5	66.0	31*	5.3	63.5	1	Straight
S. Erwee	55	93	10	2958	**200***	35.6	54.0	113	41.0	87.5	1	Straight
M. van Wyk	146	251	37	8318	**200***	38.8	68.8	175*	40.4	143.3	3	Lateral
K.A. Maharaj	74	100	19	1799	**114***	22.2	66.2	43*	13.1	87.1	3	Lateral
M. Shezi	72	86	29	546	**64**	9.5	27.4	16	6.6	43.5	1	Straight
D. M. Dupavillon	40	45	14	392	**44**	12.6	62.0	11	12.0	85.7	1	Straight

*BBTT = Batting Backlift Technique Type; Inn = Innings; Mat = Matches; NO = Not Outs; * = Not out; Highest scores = BOLD*

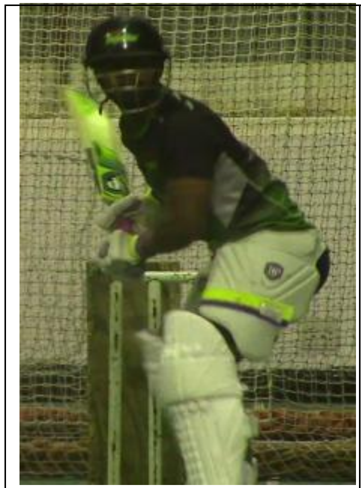	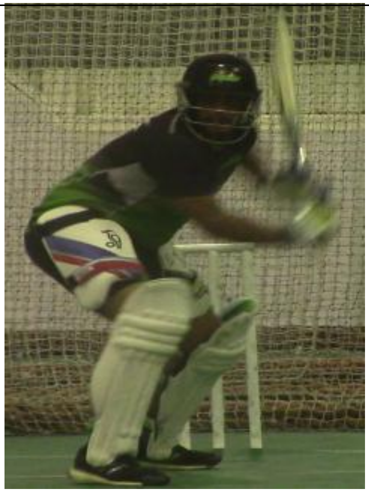	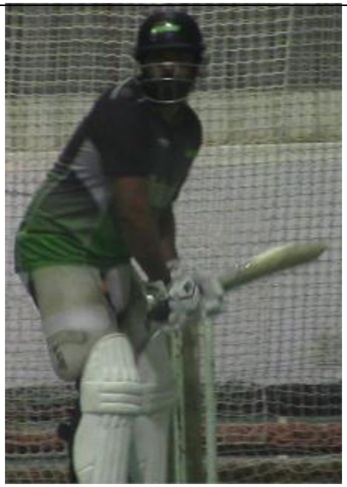	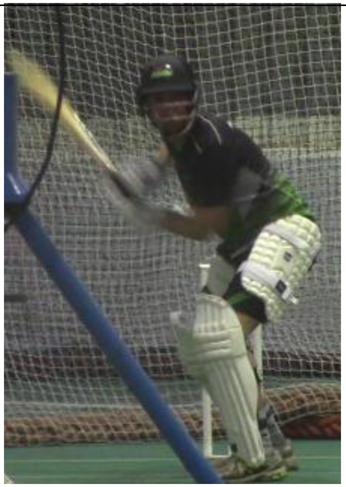	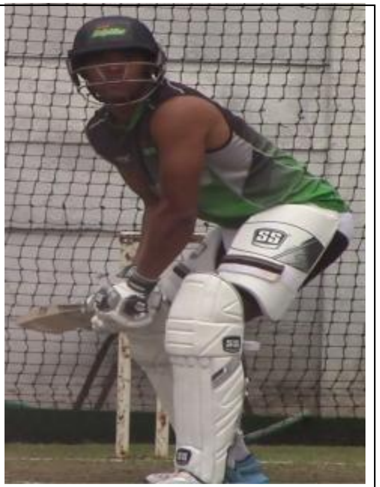
K. Zondo (S; HS = 175)	van Jaarsveld (L; HS = 160)	Vandiar (L; HS = 172*)	D. Smit (L; HS = 156*)	C. Alexander (S; HS = 54)
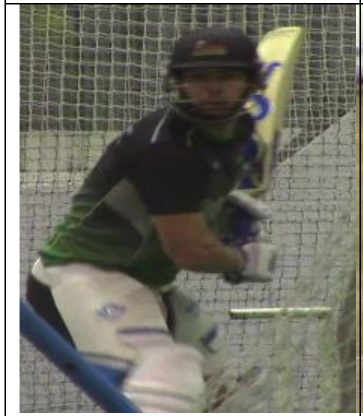	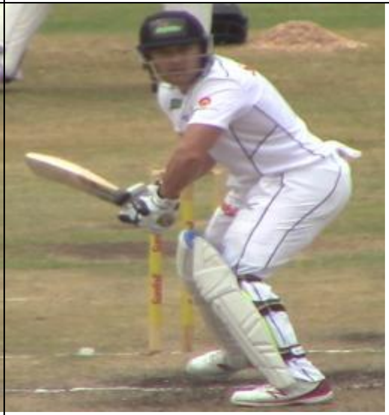	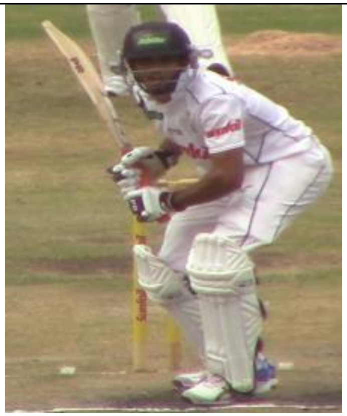	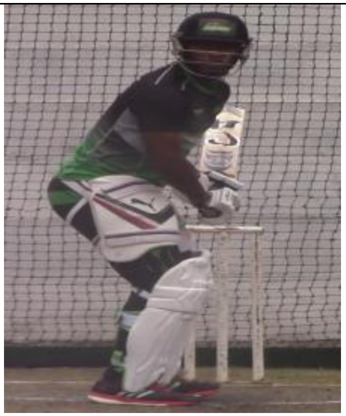	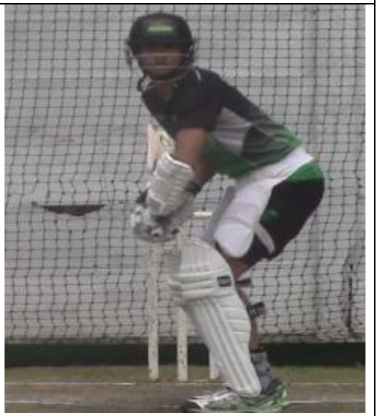
S. Erwee (S; HS = 200*)	M. van Wyk (L; HS = 200*)	Maharaj (L; HS = 114*)	M. Shezi (S; HS = 64)	Dupavillon (S; HS = 44)

Supplementary Figure 5.10: Batting backlift technique type of the Dolphins players (n = 10)

S = SBBT; L = LBBT; HS = Highest Score

Supplementary Table 19: Characteristics and performances of the Border cricket team (n = 7)

Player	Mat	Inn	NO	Runs	First Class			List A			Classifier	BBTT
					High Score	Average	Strike Rate	High Score	Average	Strike Rate		
G.V.J. Koopman	48	84	4	1981	**108**	24.7	44.0	87*	32.1	69.6	3	Lateral
S. Seyibokwe	50	84	3	2133	**101**	26.3	55.0	33	11.9	67.2	1	Straight
M. Malika	1	1	0	7	-	-	-	7	7.0	87.5	1	Straight
D.L. Brown	97	153	20	3480	**112***	26.1	48.5	76	34.9	90.7	2	Straight
J. Marais	1	2	0	30	**18**	15.0	43.4	9	5.2	47.8	2	Straight
M. Walters	57	96	7	2757	**180***	30.9	48.2	102*	34.9	67.5	3	Lateral
C.G. Bosch	2	3	0	20	13	6.6	28.5	**14**	9.5	52.7	3	Lateral

*BBTT = Batting Backlift Technique Type; Inn = Innings; Mat = Matches; NO = Not Outs; * = Not out; - = did not play Tests/ODI; Highest scores = BOLD*

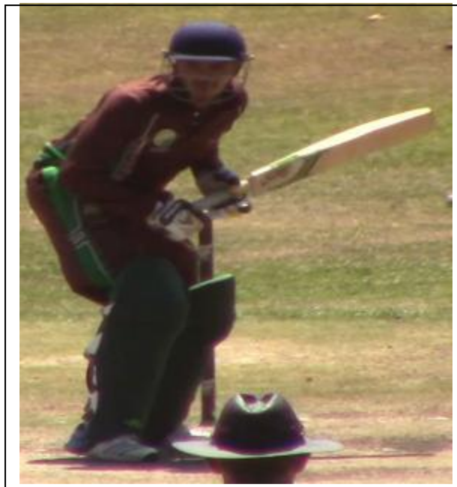	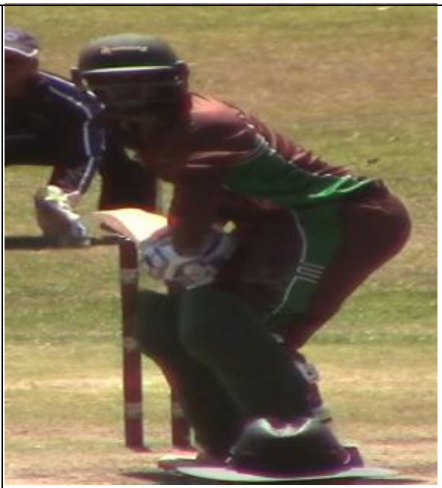	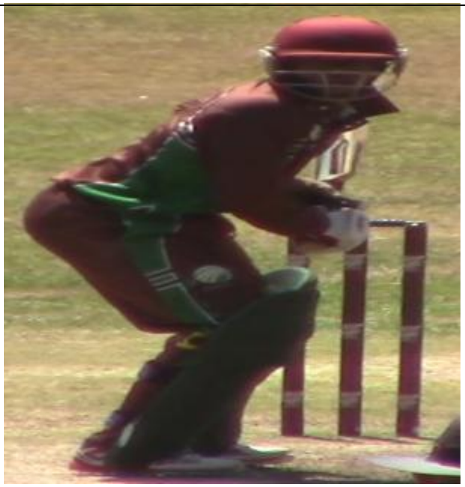	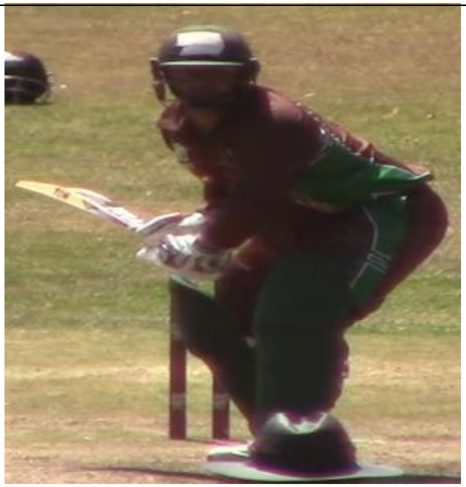
G.V.J. Koopman (L; HS = 108)	S. Seyibokwe (S; HS = 101)	M. Malika (S; HS =)	D.L. Brown (S; HS = 112*)
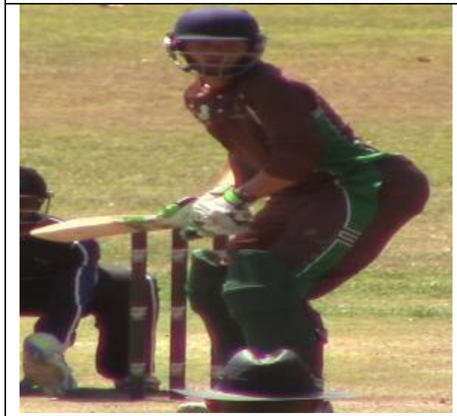	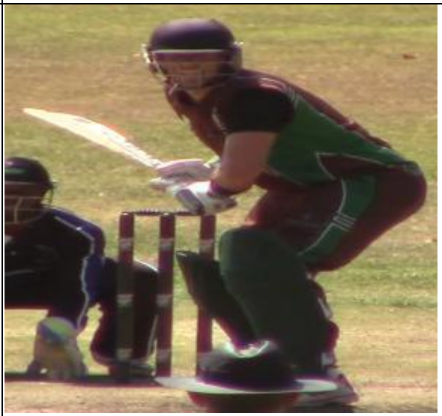	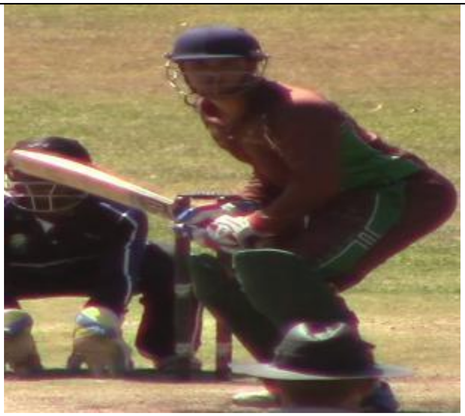	
J. Marais (S; HS = 18)	M. Walters (L; HS = 180*)	C. G. Bosch (L; HS = 14)	

Supplementary Figure 5.11: Batting backlift technique type of the Border players (n = 7)

S = SBBT; L = LBBT; HS = Highest Score

Supplementary Table 20: Characteristics and performances of the Eastern Province cricket team (n = 10)

Player	Mat	Inn	NO	Runs	First Class			List A			Classifier	BBTT
					High Score	Average	Strike Rate	High Score	Average	Strike Rate		
D.J. White	70	120	8	3528	**160**	31.5	49.3	94*	29.6	73.6	3	Lateral
E.M. Moore	36	63	8	1936	**144**	35.2	50.8	90	38.0	75.2	3	Lateral
M.C. Christensen	8	9	0	209	**84**	23.2	39.9	27	15.3	112.1	2	Straight
K.R. Smuts	74	123	8	3484	**148**	30.2	55.7	90	21.2	82.7	2	Straight
A.J.N. Price	46	71	5	2235	**167**	33.8	68.2	87	33.6	86.1	3	Lateral
O. Nyaku	12	16	1	342	**93***	22.8	43.1	30	13.5	79.4	3	Lateral
A. Nortje	19	22	8	321	**79***	22.9	55.9	16	10.0	55.5	3	Lateral
T. Bokako	24	31	2	284	**31**	9.7	39.7	28	13.3	56.3	1	Straight
E. O'Reilly	32	34	16	229	**42***	12.7	31.9	6*	4.8	45.2	2	Straight
T. Koekemoer	1	1	0	46	**46**	46.0	48.9	9	19.0	86.3	3	Lateral

*BBTT = Batting Backlift Technique Type; Inn = Innings; Mat = Matches; NO = Not Outs; * = Not out; Highest scores = BOLD*

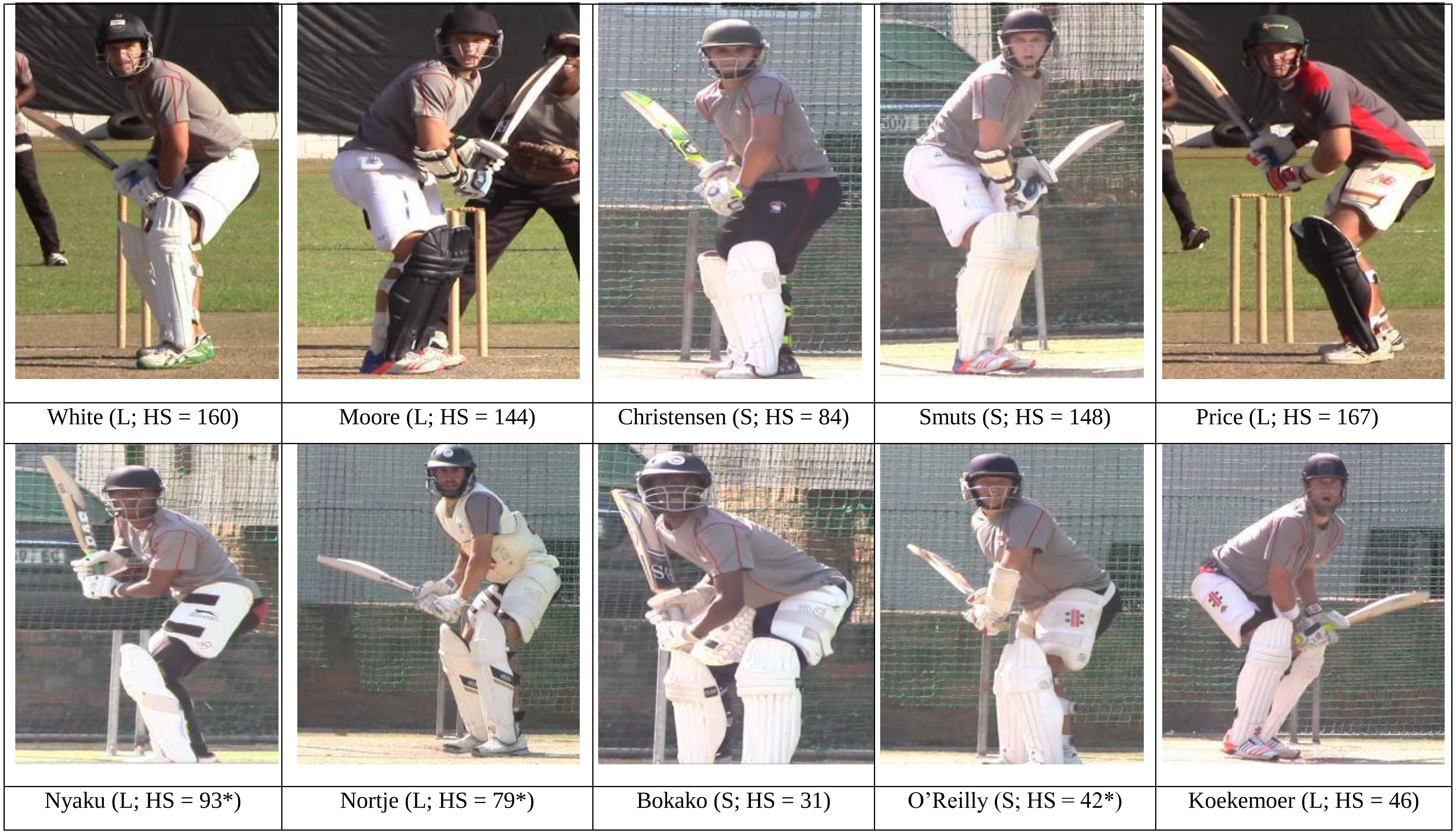

Supplementary Figure 5.12: Batting backlift technique type of the Eastern Province players (n = 10)

S = SBBT; L = LBBT; HS = Highest Score

Supplementary Table 21: Characteristics and performances of the Warriors cricket team (n = 10)

Player	Mat	Inn	NO	Runs	First Class			List A			Classifier	BBTT
					High Score	Average	Strike Rate	High Score	Average	Strike Rate		
C.N. Ackermann	61	107	11	3680	**144**	38.3	46.3	92	32.1	68.1	3	Lateral
G.L. Cloete	80	138	9	3874	**135**	30.0	49.1	85	22.7	65.8	2	Straight
M.L. Price	98	181	9	5366	**181**	31.1	51.9	155	39.7	77.6	2	Straight
J.J.T. Smuts	76	142	8	4386	**150***	32.7	61.2	132	33.8	77.9	3	Lateral
M.Y. Vallie	76	118	13	4211	**167**	40.1	58.9	96	33.0	77.5	1	Straight
M.J. Nqolo	47	72	4	1525	**137**	22.4	53.8	63	23.1	76.1	3	Lateral
C. Fortuin	18	26	2	600	72	25.0	51.0	**80**	15.0	68.2	1	Straight
S.R. Harmer	76	118	25	2450	**100***	26.3	48.7	43*	19.3	101.4	1	Straight
A. Gqamane	46	65	10	1074	86	19.5	77.7	**88***	32.6	112.7	1	Straight
S.S.B. Magala	56	80	18	1053	53	16.9	47.6	**78***	15.3	87.9	3	Lateral

*BBTT = Batting Backlift Technique Type; Inn = Innings; Mat = Matches; NO = Not Outs; * = Not out; Highest scores = BOLD*

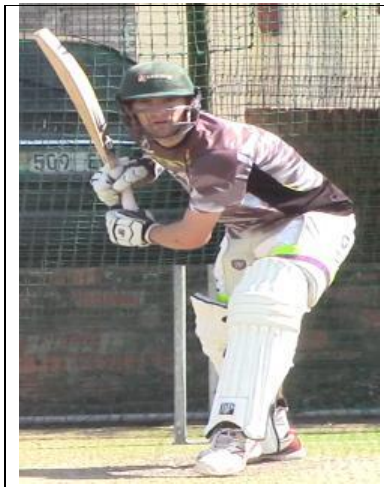	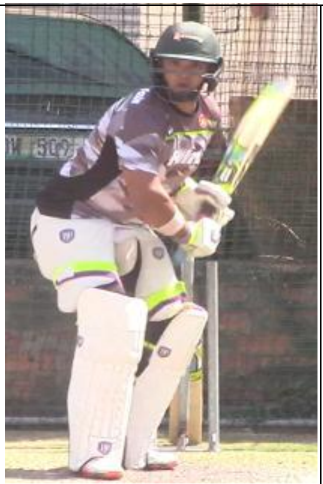	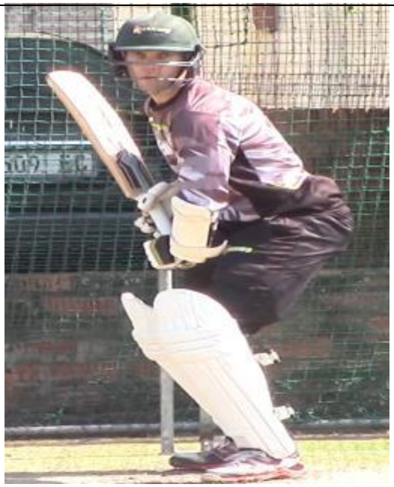	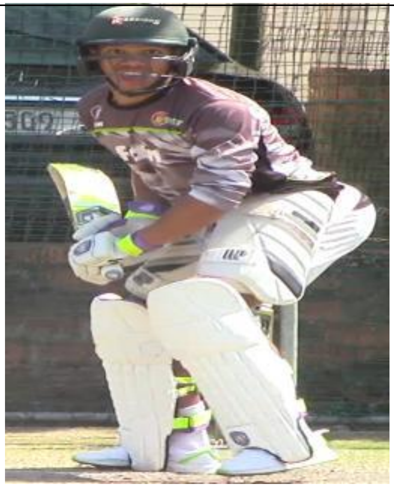	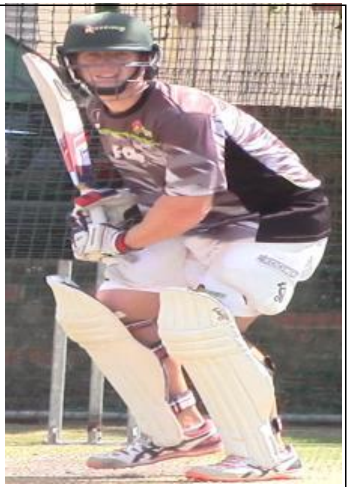
Ackermann (L; HS = 144)	Cloete (S; HS = 135)	Price (S; HS = 181)	Fortuin (S; HS = 72)	Harmer (S; HS = 100*)
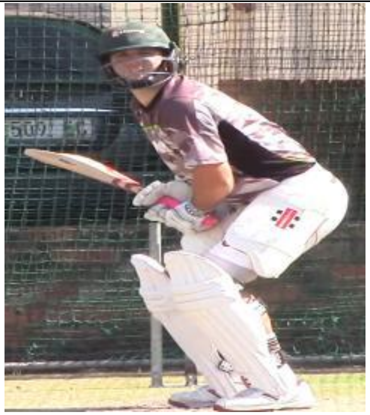	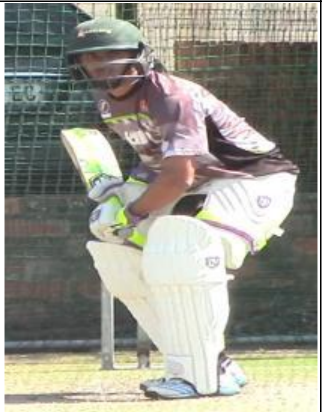	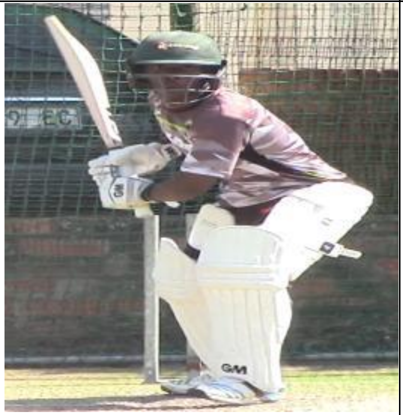	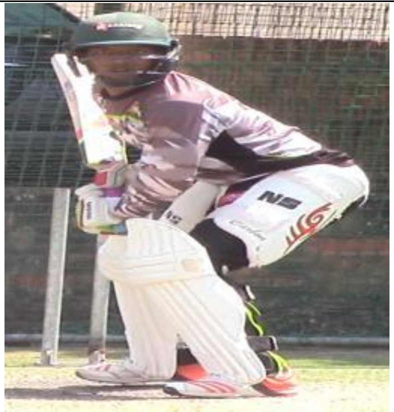	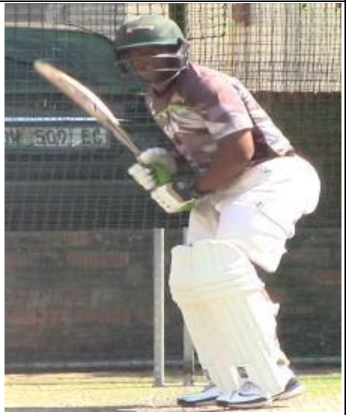
Smuts (L; HS = 150*)	Vallie (S; HS = 167)	Nqolo (L; HS = 137)	Gqamane (S; HS = 86)	Magala (L; HS = 53)

Supplementary Figure 5.13: Batting backlift technique type of the Warriors players (n = 10)

S = SBBT; L = LBBT; HS = Highest Score

Supplementary Table 22: Characteristics and performances of the Cobras cricket team (n = 8)

Player	Mat	Inn	NO	Runs	First Class			List A			Classifier	BBTT
					High Score	Average	Strike Rate	High Score	Average	Strike Rate		
A.G. Puttick	158	274	27	10057	**250***	40.7	-	143	36.3	-	1	Straight
O.A. Ramela	86	150	7	4183	**202***	29.2	42.0	106	28.0	64.6	1	Straight
S. van Zyl	11	15	2	355	101*	27.3	53.9	**114***	37.0	73.9	2	Straight
J.L. Ontong	181	288	22	10901	**166**	40.9	36.7	122	29.4	68.9	2	Straight
D.J. Vilas	88	132	14	4845	**216***	68.6	44.7	120	33.0	95.5	1	Straight
C. Tshiki	27	46	1	928	**175**	20.6	43.4	98	22.8	65.5	2	Straight
W.D. Parnell	56	75	6	1747	111*	25.3	51.5	**129**	24.5	85.4	3	Lateral
V. Philander	123	161	28	3388	**168**	25.4	46.4	79*	23.2	75.2	3	Lateral

*BBTT = Batting Backlift Technique Type; Inn = Innings; Mat = Matches; NO = Not Outs; * = Not out; - = did not play Tests/ODI; Highest scores = BOLD*

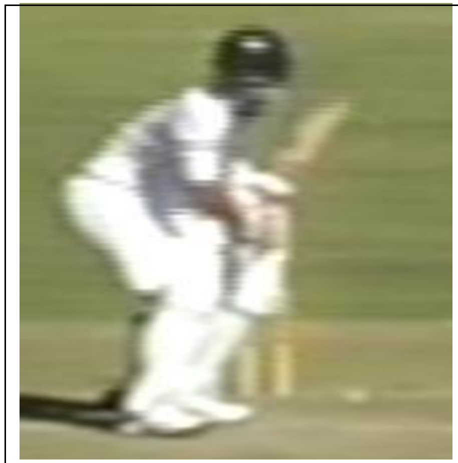	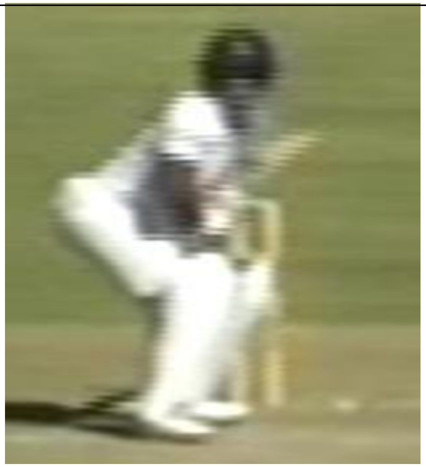	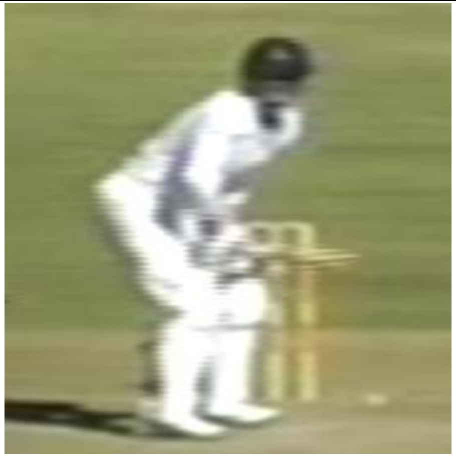	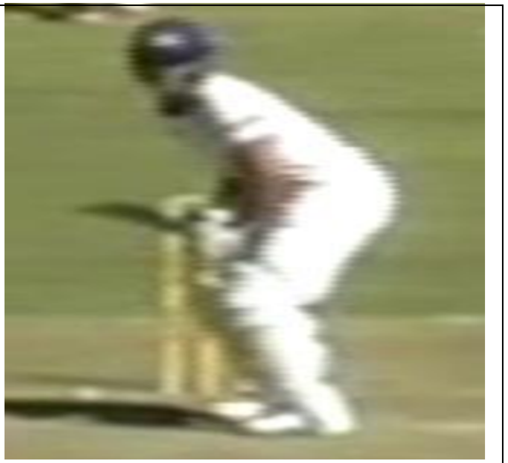
A.G. Puttick (S; HS = 250*)	O.A. Ramela (S; HS = 202*)	S. van Zyl (S; HS = 114)	J.L. Ontong (S; HS = 166)
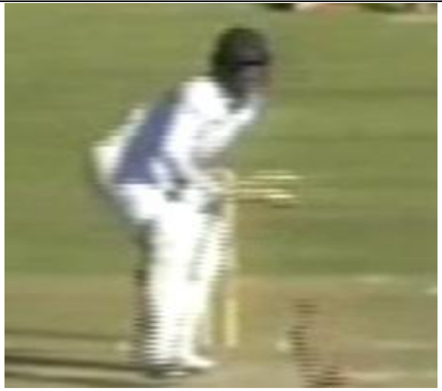	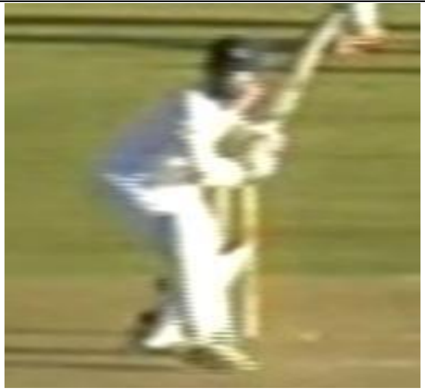	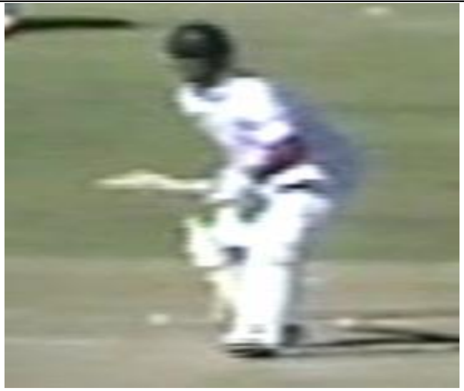	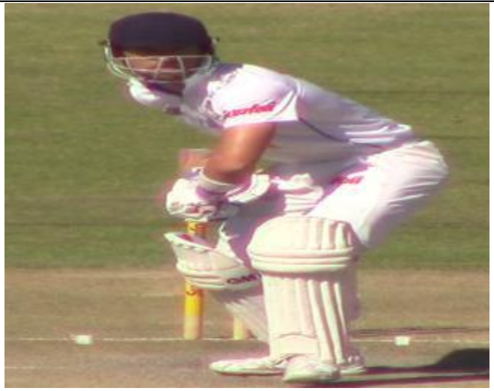
C. Tshiki (S; HS = 175)	W.D. Parnell (L; HS = 129)	V. Philander (L; HS = 168)	D.J. Vilas (S; HS = 216*)

Supplementary Figure 5.14: Batting backlift technique type of the Cobras players (n = 8)

S = SBBT; L = LBBT; HS = Highest Score

Supplementary Table 23: Characteristics and performances of the Western Province players (n =10)

Note: Players from this group provided consent for participation in the study but not for their identity to be disclosed.

Amateur Players	BBTT	Classifier	First Class		List A	
			Runs	Average	Runs	Average
Player 1	Straight	1	76	7.6	15	15.0
Player 2	**Lateral**	3	5873	**41.3**	2152	**48.9**
Player 3	Straight	2	1927	27.5	487	18.7
Player 4	**Lateral**	3	570	**51.8**	103	**34.3**
Player 5	Straight	2	3000	34.0	376	17.0
Player 6	Straight	1	802	22.9	171	11.4
Player 7	Straight	2	117	23.4	6	3.0
Player 8	Straight	1	73	7.3	46	46.0
Player 9	Straight	2	90	12.8	13	13.0
Player 10	Straight	2	167	12.8	114	16.2

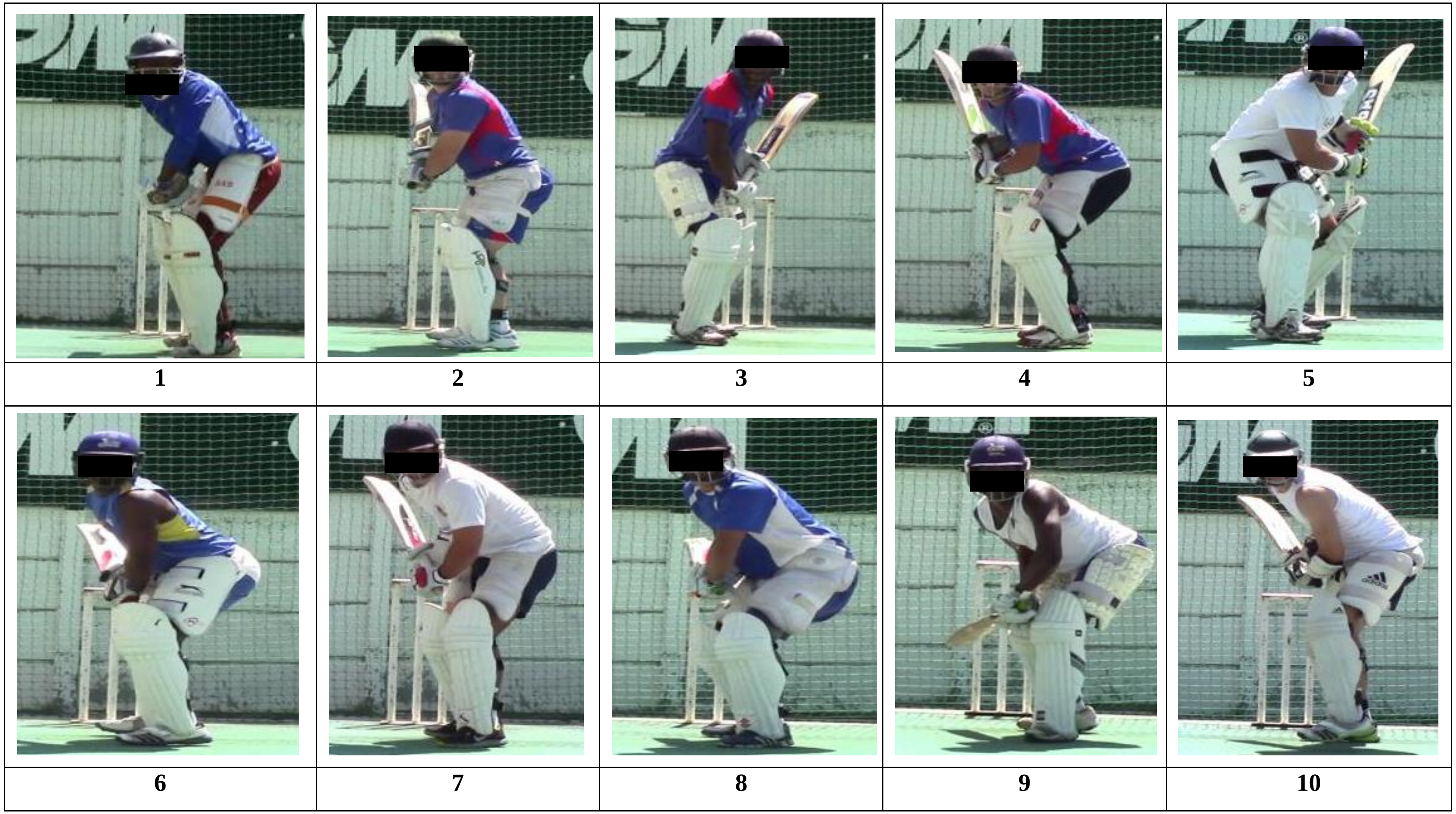

Supplementary Figure 5.15: Batting backlift technique type of the Western Province players (n = 10)

Note: This team provided consent to participate in the study but not for their identity to be disclosed.

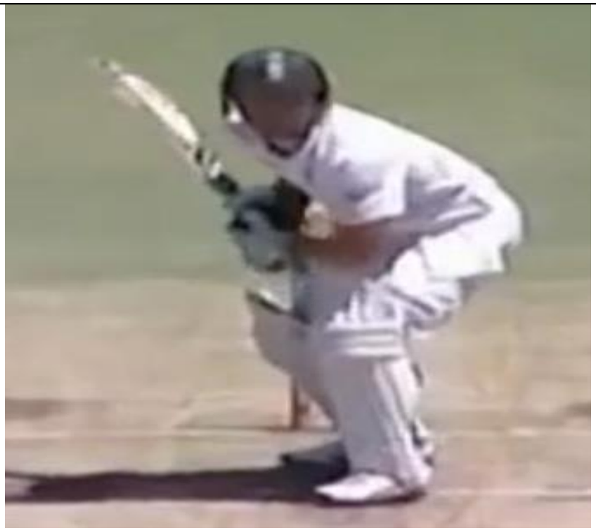	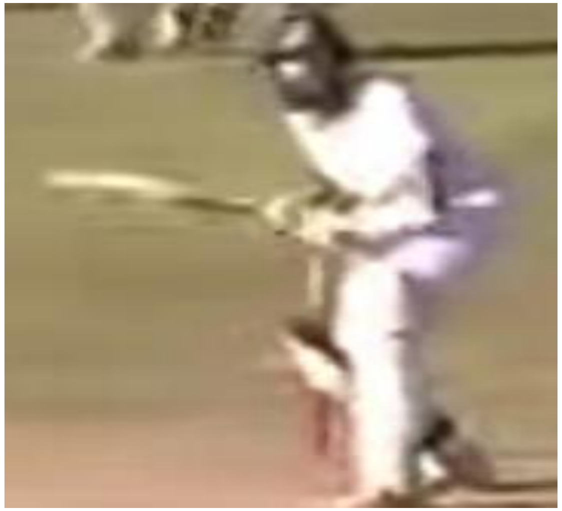	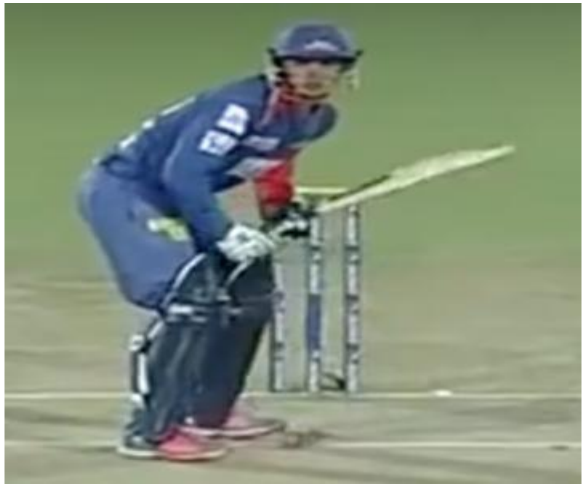
AB de Villiers (L; HS = 278*)	Hashim Amla (L; HS = 311*)	Quinton de Kock (L; HS = 129)
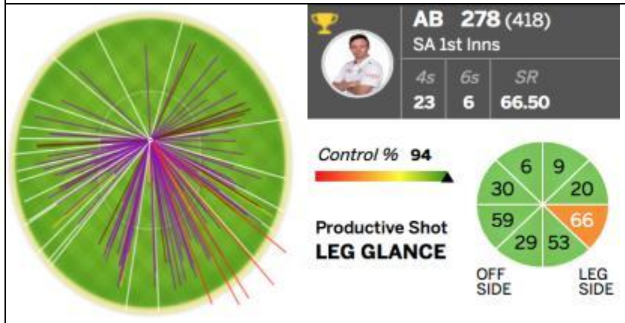	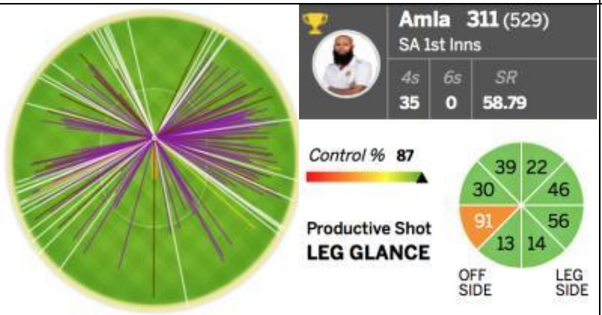	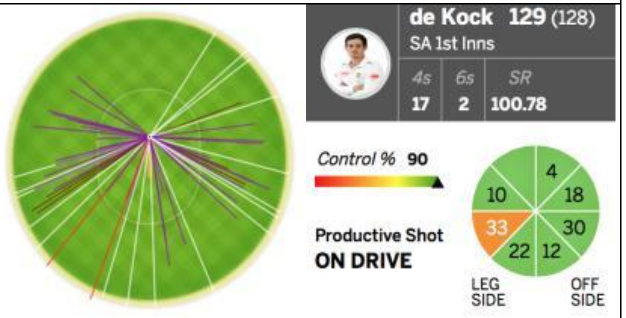

<table>
<tr>
<td>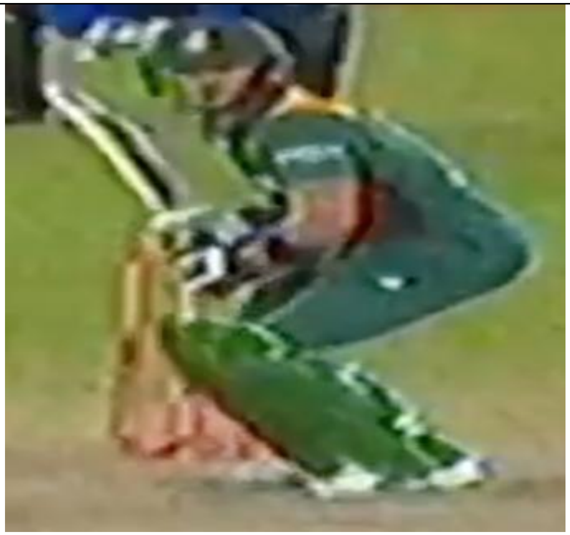</td>
<td>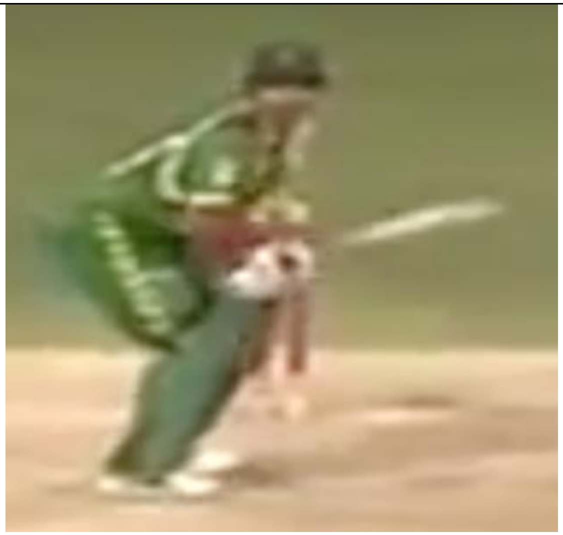</td>
<td>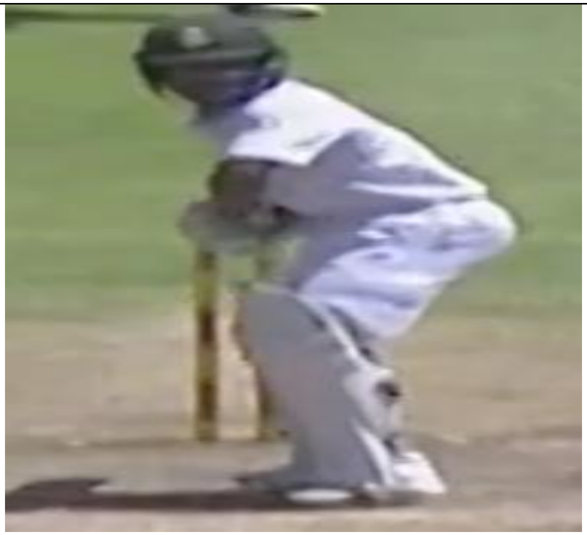</td>
</tr>
<tr>
<td>Faf du Plessis (L; HS = 137)</td>
<td>JP Duminy (L; HS = 166)</td>
<td>Temba Bavuma (S; HS = 102)</td>
</tr>
<tr>
<td>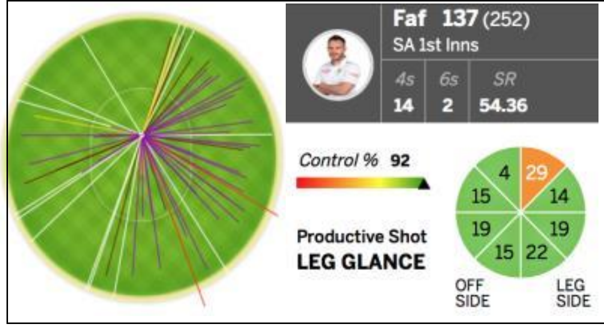
</td>
<td>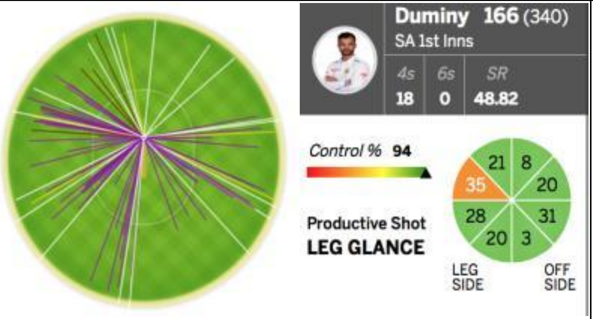
</td>
<td>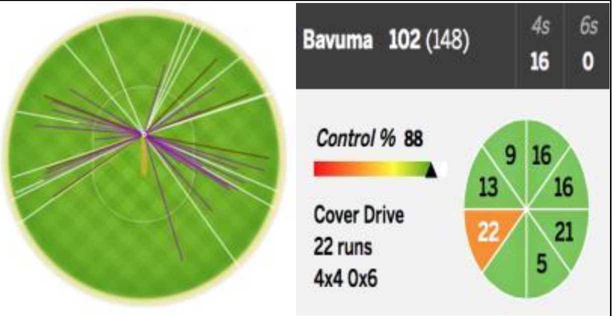
</td>
</tr>
</table>

<table>
<tr><td>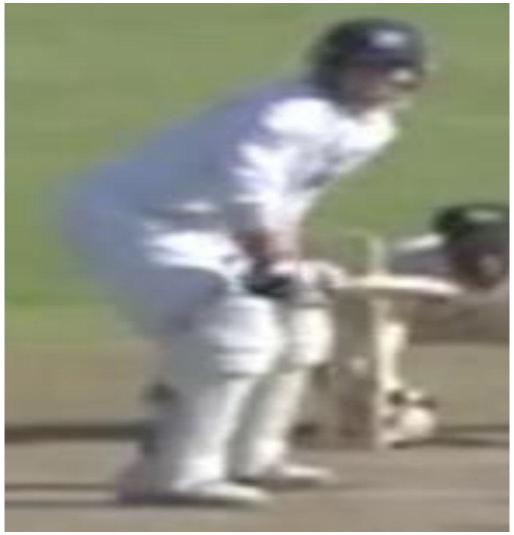</td><td>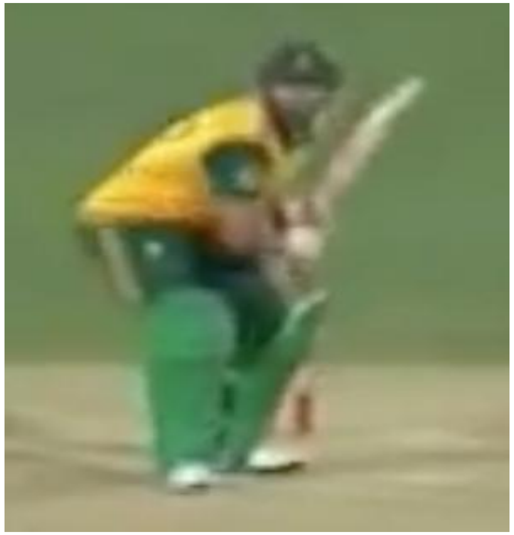</td><td>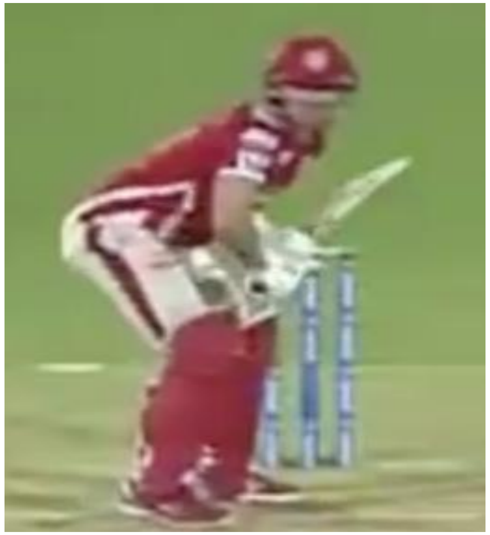</td></tr>
<tr><td>Stiaan van Zyl (S; HS = 101)</td><td>Rilee Rossouw (L; HS = 132)</td><td>David Miller (L; HS = 138)</td></tr>
<tr><td>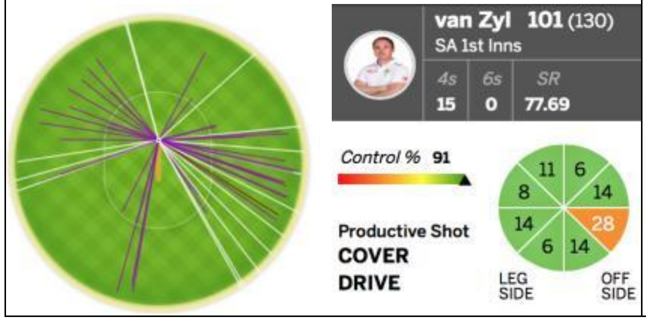
</td><td>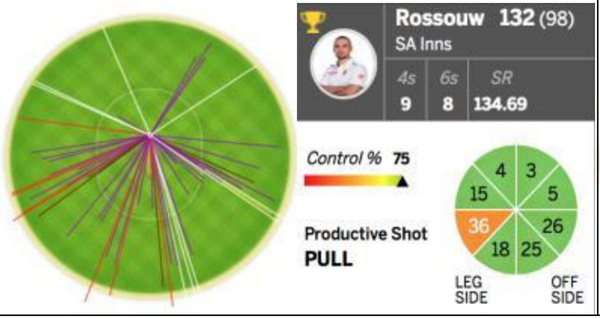
</td><td>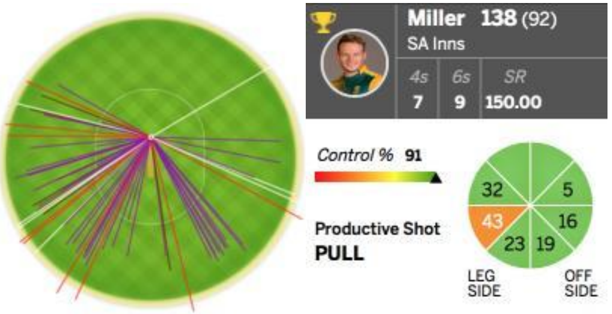
</td></tr>
</table>

<table>
<tr>
<td>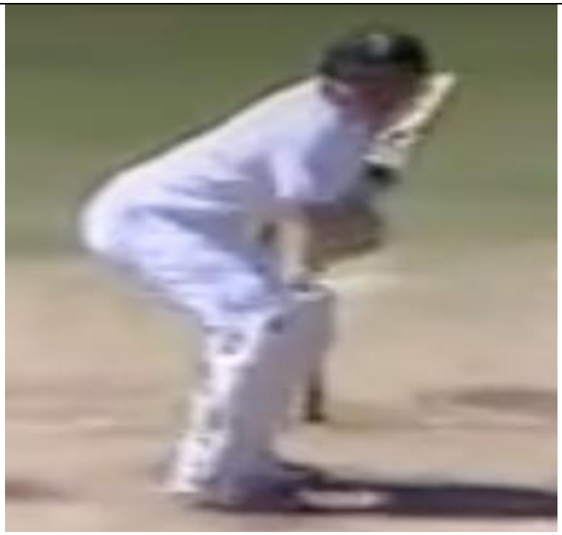</td>
<td>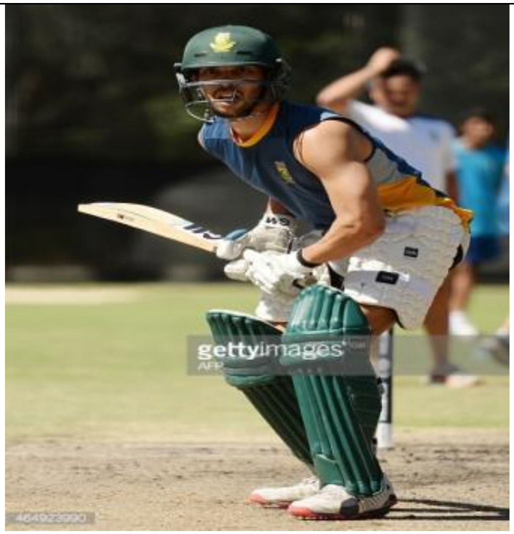
</td>
<td>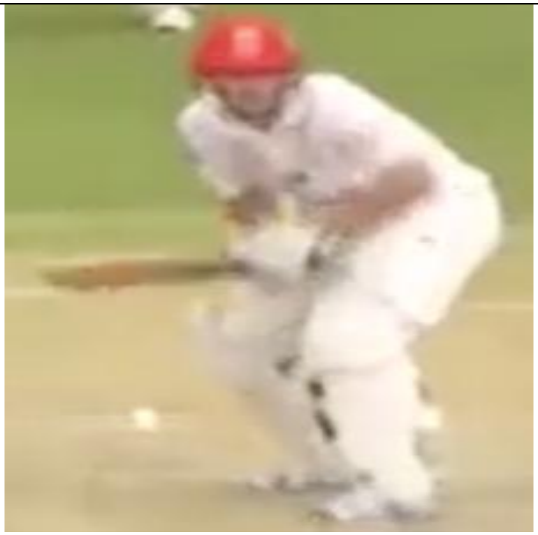</td>
</tr>
<tr>
<td>Dean Elgar (S; HS = 121)</td>
<td>Farhaan Behardien (L; HS = 70)</td>
<td>Stephen Cook (L; HS = 115)</td>
</tr>
<tr>
<td>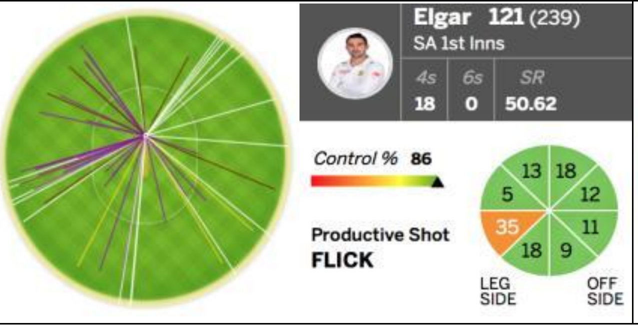
</td>
<td>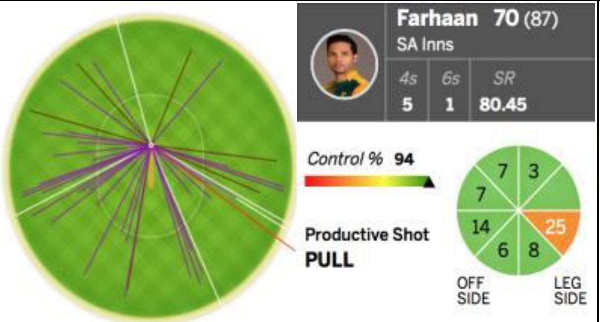
</td>
<td>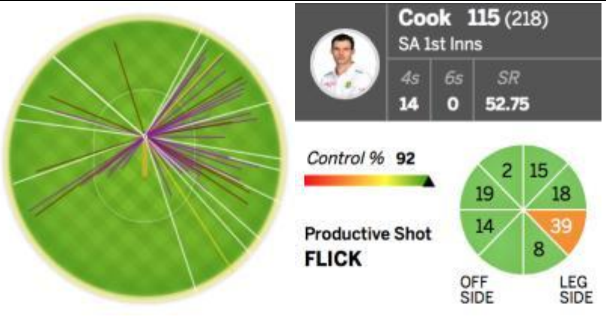
</td>
</tr>
</table>

Supplementary Figure 5.16: South African batsmen's highest score at Test or ODI levels (n = 12)

HS = Highest Score in their Test/ODI career; L = Lateral batting backlift technique; S = Straight batting backlift technique; % = percentage; Inns = innings; 1st = first; 4s = fours; 6s = sixes; SR = strike rate;
